# Novel family of nitrogen-rich energetic (1,2,4-triazolyl) furoxan salts with balanced performance

**DOI:** 10.3389/fchem.2022.1012605

**Published:** 2022-09-12

**Authors:** Alexander A. Larin, Alla N. Pivkina, Ivan V. Ananyev, Dmitry V. Khakimov, Leonid L. Fershtat

**Affiliations:** ^1^ N. D. Zelinsky Institute of Chemistry, Russian Academy of Sciences, Moscow, Russia; ^2^ Department of Chemistry, National Research University Higher School of Economics, Moscow, Russia; ^3^ N.N. Semenov Federal Research Centre for Chemical Physics, Russian Academy of Sciences, Moscow, Russia; ^4^ N.S. Kurnakov Institute of General and Inorganic Chemistry, Russian Academy of Sciences, Moscow, Russia; ^5^ A.N. Nesmeyanov Institute of Organoelement Compounds, Russian Academy of Sciences, Moscow, Russia

**Keywords:** nitrogen heterocycle, energetic material, energetic salts, 1,2,4-triazoIe, furoxan

## Abstract

Nitrogen-rich energetic materials comprised of a combination of several heterocyclic subunits retain their leading position in the field of materials science. In this regard, a preparation of novel high-energy materials with balanced set of physicochemical properties is highly desired. Herein, we report the synthesis of a new series of energetic salts incorporating a (1,2,4-triazolyl) furoxan core and complete evaluation of their energetic properties. All target energetic materials were well characterized with IR and multinuclear NMR spectroscopy and elemental analysis, while compound **6** was further characterized by single-crystal X-ray diffraction study. Prepared nitrogen-rich salts have high thermal stability (up to 232°C), good experimental densities (up to 1.80 g cm^−3^) and high positive enthalpies of formation (344–1,095 kJ mol^−1^). As a result, synthesized energetic salts have good detonation performance (*D* = 7.0–8.4 km s^−1^; *p* = 22–32 GPa), while their sensitivities to impact and friction are quite low.

## 1 Introduction

Functional organic materials are one of the most important and emerging area of research in state-of-the-art materials science (Li and Yu, 2021; Liu et al., 2021). Construction of potential high-performance materials as well as their essential components is needed to be achieved according to modern requirements of sustainable and environmentally-focused society. This issue is still relevant in the field of energetic materials science (Fershtat and Makhova, 2020; O’Sullivan and Zdilla, 2020; Zhou et al., 2022). Performance and sensitivity of currently used high-energy compounds (TNT: 2,4,6-trinitrotoluene, RDX: 1,3,5-trinitro-1,3,5-triazinane, PETN: pentaerythritol tetranitrate) are usually unbalanced, while their ecological and toxicological profiles are unfavorable. In this regard, preparation of novel eco-friendly energetic materials with high nitrogen content and acceptable safety requirements remains urgent (Kuchurov et al., 2017; Zlotin et al., 2021).

**FIGURE 1:**
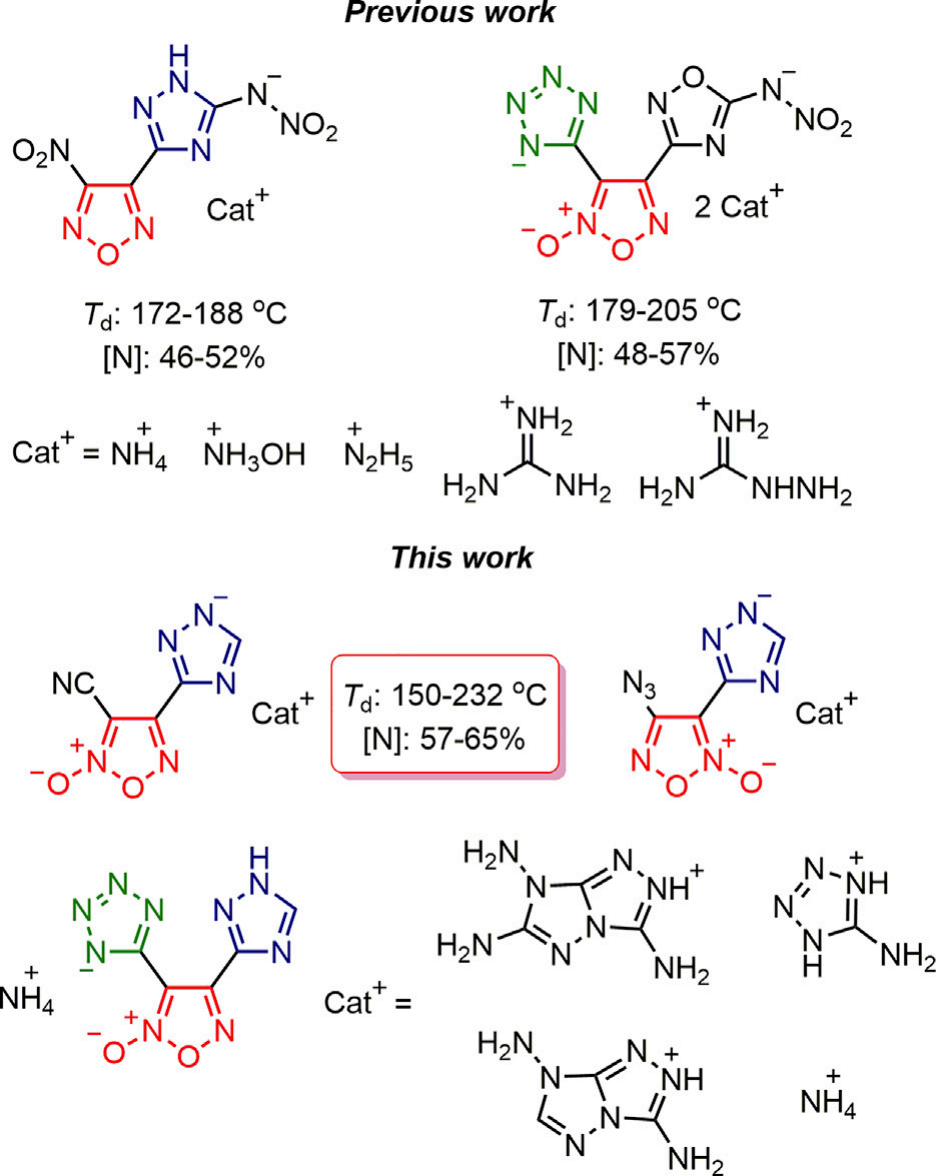
Selected examples of bi- and triheterocyclic energetic materials.

In recent decade, synthesis of organic energetic materials is often performed through a combination of various polynitrogen and nitrogen-oxygen heterocyclic scaffolds: tetrazole-furazan (Liang et al., 2012; Huang et al., 2012; Liang et al., 2013), tetrazole-furoxan (Fershtat et al., 2015a; Larin et al., 2019), 1,2,4-triazole-furazan (Xu et al., 2018; Ma et al., 2019a; Ma et al., 2019b; Sheremetev et al., 2019; Aleksandrova et al., 2020; Ma et al., 2020; Chinnam et al., 2021), tetrazole-furoxan-1,2,4-oxadiazole (Liu et al., 2018) (Figure 1). A combination of structurally diversed nitrogen-rich heterocycles linked via *N*, *N*′-ethylene bridges was also reported as one of the promising approaches to balance energetic properties of the resulted energy-rich materials (Kumar et al., 2016a; Kumar et al., 2016b). Recently, our team reported a preparation of energetic (1,2,4-triazolyl) furoxans with good detonation performance and decreased sensitivity (Larin et al., 2022). However, it is known that NH-fragment of the 1,2,4-triazole ring is weakly acidic (Garratt, 1996) and may stimulate corrosivity which is undesired for any potential application. Although examples on the preparation of energetic salts bearing a 1,2,4-triazolate anion are rather scarce (except NTO-based those) (Parisi et al., 2021; Creegan et al., 2022), such strategy may serve as a convenient solution to overcome this issue. High-energy salts usually have acceptable thermal stability (>150°C) and moderate sensitivity to mechanical stimuli, while owing to the high nitrogen content these metal-free salts have a high level of environmental compatibility (Larin et al., 2019; Larin and Fershtat, 2021). Herein, we present the synthesis of a new series of energetic salts comprising a (1,2,4-triazolyl) furoxan core (Figure 1) and complete evaluation of their energetic properties.

## 2 Materials and methods

**CAUTION!** Although we have encountered no difficulties during preparation and handling of the compounds described in this paper, they are potentially explosive energetic materials that are sensitive to impact and friction. Mechanical actions of these energetic materials, involving scratching or scraping, must be avoided. Any manipulations must be carried out by using appropriate standard safety precautions.

All reactions were carried out in well-cleaned oven-dried glassware with magnetic stirring. ^1^H and ^13^C NMR spectra were recorded with a Bruker AM-300 (300.13 and 75.47 MHz, respectively) spectrometer and referenced to the residual solvent peak. ^14^N NMR spectra were measured with a Bruker AM-300 (21.69 MHz) spectrometer by using MeNO_2_ [*δ*(^14^N) = 0.0 ppm] as an external standard. ^15^N NMR spectra were recorded with a Bruker DRX500 instrument (the frequency for ^15^N was 50.7 MHz) at room temperature. The chemical shifts are reported in ppm (*δ*). Coupling constants, *J*, are reported in Hertz. The IR spectra were recorded with a Bruker “Alpha” spectrometer in the range 400–4,000 cm^−1^ (resolution 2 cm^−1^). Elemental analyses were performed by the CHN Analyzer PerkinElmer 2,400. The melting points were determined with a Stuart SMP20 apparatus and are uncorrected. All standard reagents were purchased from Aldrich or Acros Organics and used without further purification. Amidrazones **2a** (Fershtat et al., 2015b) and **2b** (Larin et al., 2021), 3-cyano-4-(1*H*-1,2,4-triazol-3-yl)furoxan **3a** and 4-azido-3-(1*H*-1,2,4-triazol-3-yl)furoxan **3b** (Larin et al., 2022) were synthesized according to the literature.

### 2.1 X-ray diffraction study

The colorless single crystals of **6** were grown from DMF. At 100 K crystals of **6** (C_5_H_6_N_10_O_2_) are triclinic, space group P-1: a = 4.8372(2), b = 9.6045(4), c = 10.1054(4) Å, α = 94.895(3), β = 92.103(2), γ = 98.814(2), V = 461.66(3) Å^3^, Z = 2, d_calc_ = 1.714 g cm^−3^, F(000) = 244. Intensities of 4,822 reflections were measured with a Bruker APEX II CCD diffractometer [λ(MoKα) = 0.71073 Å, ω-scans, 2θ < 58°] and 2,198 independent reflections [R_int_ = 0.0336] were used in further refinement. The structure was solved by direct method and refined by the full-matrix least-squares technique against F^2^ in the isotropic-anisotropic approximation. The hydrogen atoms were found in difference Fourier synthesis and refined in the isotropic approximation. For **6**, the refinement converged to wR2 = 0.1207, GOOF = 0.992 and R1 = 0.0429 for 1,602 independent reflections with I > 2σ(I). All calculations were performed using SHELXL-2014/6 (Sheldrick, 2015). CCDC 2193015 contains the supplementary crystallographic data for **6**·H_2_O. These data can be obtained free of charge via http://www.ccdc.cam.ac.uk/conts/retrieving.html (or from the CCDC, 12 Union Road, Cambridge, CB21EZ, UK; or deposit@ccdc.cam.ac.uk).

### 2.2 Computational methods

All DFT calculations for **6**, TILMEI and DUQGUT were performed with the Gaussian09 program (Frisch et al., 2016). The optimization procedures of all model structures (together with the optimization of hydrogen atoms positions for the isolated ions in crystal geometries) are done invoking the standard cutoff criteria and using the def2-TZVP basis set (Weigend and Ahlrichs, 2005; Weigend, 2006) and the PBE0 functional (Perdew et al., 1996; Adamo and Barone, 1999) with the Grimme et al. (2010) empirical dispersion correction and Becke-Johnson damping (Grimme et al., 2011). The non-specific solvation effects are modelled for **6** by means of the self-consistent reaction field within the polarizable continuum model (integral equation formalism, ε = 72, water) (Tomasi et al., 2005). According to the calculation of energy second derivatives (ultrafine grids), all fully optimized structures correspond to minima on potential energy surface.

The Δ_OED_ analysis for **6**, TILMEI and DUQGUT were done based on the HF/3-21G electron densities calculated for the crystal fragments and the PBE0/def2-TZVP electron densities calculated for the isolated ions in crystal geometries. The X-H bond lengths for all these calculations were fixed on the values obtained upon the partial optimization of isolated ions in crystal geometries. The crystal fragments contain 24 anions and 24 cations for the DUQGUT structure, 23 anions and 23 cations for the TILMEI structure and 22 anions and 22 cations for **6**. The partitioning of real space into atoms was done within the “Atoms in Molecules” theory. The volume integration (both, over the atomic basins for the central molecule in a cluster and over the volume enclosed by 0.0004 a.u. isosurface of electron density for isolated ions) was performed using the Multiwfn program (Lu and Chen, 2012). The SHELX (Sheldrick, 2015) atomic mass table was used to calculate density values.

The heat of formation calculations were carried out combining the atomization energy method (Eq. 1) (Ochterski et al., 1996; Montgomery et al., 2000) with CBS-4M electronic enthalpies (Klapötke, 2012; Khakimov et al., 2015). CBS-4M energies of the atoms were calculated with the Gaussian 09 software package (Frisch et al., 2016). Values for Δ_f_ *H*° (atoms) were taken from the NIST database.

$$\Delta_{f}H^{\circ }\left(\mathrm{g,298}\right)=H\left(\mathrm{Molecule,298}\right)-\sum H^{\circ }\left(\mathrm{Atoms,298}\right)+\sum \Delta_{f}H^{\circ }\left(\mathrm{Atoms,298}\right)$$ (1)

Geometric optimization of all structures for crystal packing calculation was carried out using the DFT/B3LYP functional and the aug-cc-PVDZ basis set with a Grimme (2011) D2 dispersion correction. The optimized structures were conformed to be true local energy minima on the potential-energy surface by frequency analyses at the same level.

In the calculation of lattice energy, the molecules were treated as rigid bodies with foxed point groups. We apply pairwise atom-atom potentials to describe the van der Waals and electrostatic point charges for Coulomb components of intermolecular energy. At the initial stage ‘6–12’ Lennard-Jones (LJ) type potential parameters were used (Momany et al., 1974). The electrostatic energy was calculated with a set of displaced point charge sites by program FitMEP (Dzyabchenko, 2008a). The lattice energy simulations were performed with the program PMC (Dzyabchenko, 2008b).

It is well known that the majority of organic crystal structures studied experimentally belong to a rather limited number of space groups (Belsky et al., 1995). For brief assessment of crystal packing, we get the following ordered list of the most likely structural classes: P2_1_/c, P2_1_2_1_2_1_, P-1, Pca2_1_ and P1 with two independent molecules in cell, which cover more than 80% of the whole number of crystal structures in total (Belsky et al., 1995). Taking into account low deviation in crystal lattice energies of polymorphs, such calculation is considered reasonable.

Enthalpies of sublimation for **1a** and **1b** were calculated by formula:

$$\Delta H_{\mathrm{sub1}}=-E_{\mathrm{1at}}-2\mathrm{RT,}$$

where R is the universal gas constant, E_lat_ is the lattice energy, T is temperature (298 K).

The new approach for salts proposes a technique based on modeling the crystal packing for a salt and a similar neutral compound (quasi-salt, cocrystal). The enthalpy of formation in this case is calculated as the average value between these two structures (Khakimov and Pivina, 2022).

Detonation performance parameters (detonation velocity at maximal density and Chapman-Jouguet pressure) were calculated by recently suggested set of empirical methods from PILEM application (Muravyev et al., 2022). Note, that the accuracy of the utilized in PILEM empirical methods is comparable to benchmark thermodynamic code EXPLO5.

### 2.3 Thermal analysis and sensitivity measurements

Thermal analysis of the substances was carried out with a Netzsch DSC 204 HP apparatus. Samples (0.2–3.0 mg, depending on their heat release rate) were placed in aluminum pans covered with pierced lids. The samples were heated up to 400°C at a constant rate of 5 K min^−1^. Impact sensitivity tests were performed by using a BAM-type fallhammer according to STANAG 4489 standard. The reported values (IS) are the drop energies corresponding to 50% probability of explosion obtained by Bruceton analysis. Friction sensitivity was evaluated in agreement with STANAG 4487 standard. The reported quantity (FS) is the friction force corresponding to 50% probability of explosion. The details of safety test procedures can be found elsewhere (Muravyev et al., 2021).

### 2.4 Syntheses

**General procedure for the preparation of (1,2,4-triazolyl)furoxans silver salts 3a,b.** A solution of AgNO_3_ (2.04 g, 12 mmol) in H_2_O (50 ml) was added dropwise to a magnetically stirred solution of the corresponding 1*H*-1,2,4-triazol-3-yl-furoxan **1a,b** (12 mmol) in MeOH (45 ml). The resulting mixture was stirred at ambient temperature for 5 h, the solid formed was filtered off, washed with water (3 × 100 ml) and dried in air.

**Silver 3-(4-azido-2-oxido-1,2,5-oxadiazol-3-yl)-1,2,4-triazol-1-ide (3a)**: Yield 3.38 g (94%); white solid; elemental analysis calcd (%) for C_4_HAgN_8_O_2_ (299.93): C 15.96, H 0.33, N 37.23; found: C 16.10, H 0.28, N 36.97.

**Silver 3-(3-cyano-2-oxido-1,2,5-oxadiazol-4-yl)-1,2,4-triazol-1-ide (3b)**: Yield 3.01 g (88%); white solid; elemental analysis calcd (%) for C_5_HAgN_6_O_2_ (283.92): C 21.07, H 0.35, N 29.49; found: C 20.90, H 0.47, N 29.12.

**General procedure for the preparation of energetic salts 4a-c, 5a-c**: Corresponding chloride (2 mmol) was added to a magnetically stirred suspension of the silver salt **3a,b** (2 mmol) in H_2_O (30 ml) at 20 C. The resulting mixture was stirred at 60°C for 24 h. Then AgCl was removed by filtration and the solvent was evaporated *in vacuo*. The residue was recrystallized from water and dried in a vacuum desiccator over P_2_O_5_ for 24 h.

**3,6,7-Triamino-7*H*-[1,2,4]triazolo[4,3-*b*][1,2,4]triazol-2-ium 3-(4-azido-2-oxido-1,2,5-oxadiazol-3-yl)-1,2,4-triazol-1-ide (4a)**: Yield 0.54 g (78%); red solid; ^1^H NMR (300 MHz, DMSO-*d*_6_): *δ* = 8.87 (s, 1H), 6.79 (s, 2H), 6.72 (s, 2H), 5.69 (s, 2H); ^13^C{^1^H} NMR (75.5 MHz, DMSO-*d*_6_): *δ* = 159.7, 153.1, 148.7, 147.1, 146.0, 142.9, 105.5; ^14^N NMR (21.7 MHz, DMSO-*d*_6_): *δ* = −147.6 (s, N_3_); IR (KBr): 3,418, 3,306, 3,137, 2,163, 1,699, 1,634, 1,572, 1,545, 1,414, 1,385, 1,267, 1,231, 1,199, 985 cm^−1^; elemental analysis calcd (%) for C_7_H_8_N_16_O_2_ (348.10): C 24.14, H 2.32, N 64.35; found: C 23.91, H 2.46, N 64.09; IS: 7.8 J, FS: >360 N.

**5-Amino-1*H*-tetrazol-4-ium 3-(4-azido-2-oxido-1,2,5-oxadiazol-3-yl)-1,2,4-triazol-1-ide (4b)**: Yield 0.42 g (75%); dark yellow solid; ^1^H NMR (300 MHz, DMSO-*d*_6_): *δ* = 8.86 (s, 1H); ^13^C{^1^H} NMR (75.5 MHz, DMSO-*d*_6_): *δ* = 156.6, 153.1, 147.1, 146.0, 105.5; ^14^N NMR (21.7 MHz, DMSO-*d*_6_): *δ* = −148.2 (s, N_3_); IR (KBr): 3,445, 3,136, 2,164, 1,633, 1,546, 1,519, 1,413, 1,384, 1,267, 1,233, 1,199, 1,022, 985 cm^−1^; elemental analysis calcd (%) for C_5_H_5_N_13_O_2_ (279.07): C 21.51, H 1.81, N 65.22; found: C 21.28, H 1.97, N 64.96; IS: 9 J, FS: 360 N.

**3,7-Diamino-7*H*-[1,2,4]triazolo[4,3-b][1,2,4]triazol-2-ium 3-(4-azido-2-oxido-1,2,5-oxadiazol-3-yl)-1,2,4-triazol-1-ide (4c)**: Yield 0.53 g (80%); orange solid; ^1^H NMR (300 MHz, DMSO-*d*_6_): *δ* = 14.68 (br. s, 1H), 8.86 (s, 1H), 8.52 (s, 1H), 6.85 (s, 2H), 6.11 (s, 2H); ^13^C{^1^H} NMR (75.5 MHz, DMSO-*d*_6_): *δ* = 153.1, 151.4, 149.2, 147.1, 146.0, 143.4, 105.5; ^14^N NMR (21.7 MHz, DMSO-*d*_6_): *δ* = −146.4 (s, N_3_); IR (KBr): 3,440, 3,313, 3,102, 2,163, 1,698, 1,642, 1,590, 1,539, 1,412, 1,384, 1,266, 1,230, 1,199, 1,001, 957, 751 cm^−1^; elemental analysis calcd (%) for C_7_H_7_N_15_O_2_ (333.09): C 25.23, H 2.12, N 63.05; found: C 25.46, H 1.98, N 62.81; IS: 5 J, FS: 260 N.

**3,6,7-Triamino-7*H*-[1,2,4]triazolo[4,3-*b*][1,2,4]triazol-2-ium 3-(3-cyano-2-oxido-1,2,5-oxadiazol-4-yl)-1,2,4-triazol-1-ide (5a)**: Yield 0.55 g (83%); light orange solid; ^1^H NMR (300 MHz, DMSO-*d*_6_): *δ* = 8.96 (s, 1H), 6.66 (s, 2H), 6.35 (s, 2H), 5.65 (s, 2H); ^13^C{^1^H} NMR (75.5 MHz, DMSO-*d*_6_): *δ* = 159.9, 149.7, 149.1, 148.6, 146.6, 142.6, 107.2, 98.3; IR (KBr): 3,417, 3,330, 3,304, 2,253, 1,699, 1,641, 1,620, 1,538, 1,509, 1,452, 1,385, 1,346, 1,277, 1,056, 971, 832 cm^−1^; elemental analysis calcd (%) for C_8_H_8_N_14_O_2_ (332.10): C 28.92, H 2.43, N 59.02; found: C 28.69, H 2.61, N 58.78; IS: 19 J, FS: 220 N.

**5-Amino-1*H*-tetrazol-4-ium 3-(3-cyano-2-oxido-1,2,5-oxadiazol-4-yl)-1,2,4-triazol-1-ide (5b)**: Yield 0.47 g (90%); yellow solid; ^1^H NMR (300 MHz, DMSO-*d*_6_): *δ* = 8.93 (s, 1H), 6.43 (s, 2H); ^13^C{^1^H} NMR (75.5 MHz, DMSO-*d*_6_): *δ* = 156.5, 149.6, 149.1, 146.5, 146.2, 107.2, 98.2; IR (KBr): 3,452, 3,147, 2,252, 2,164, 1,632, 1,540, 1,452, 1,385, 1,274, 1,250, 1,058, 970, 832 cm^−1^; elemental analysis calcd (%) for C_6_H_5_N_11_O_2_ (263.06): C 27.38, H 1.92, N 58.54; found: C 27.21, H 2.03, N 58.31; IS: 21 J, FS: 250 N.

**3,7-Diamino-7*H*-[1,2,4]triazolo[4,3-*b*][1,2,4]triazol-2-ium 3-(3-cyano-2-oxido-1,2,5-oxadiazol-4-yl)-1,2,4-triazol-1-ide (5c)**: Yield 0.49 g (78%); dark yellow solid; ^1^H NMR (300 MHz, DMSO-*d*_6_): *δ* = 8.96 (s, 1H), 8.57 (s, 1H), 7.13 (s, 2H), 6.14 (s, 2H), 5.79 (s, 2H); ^13^C{^1^H} NMR (75.5 MHz, DMSO-*d*_6_): *δ* = 151.4, 149.7, 149.2, 149.1, 146.6, 143.4, 107.2, 98.3; ^15^N NMR (60.8 MHz, DMSO-*d*_6_): *δ* = −3.6, −9.7, −11.3, −18.4, −94.6, −131.6, −136.2, −172.6, −172.8, −185.2, −242.8, −317.5, −328.7; IR (KBr): 3,313, 3,280, 3,103, 1700, 1,645, 1,622, 1,590, 1,536, 1,427, 1,384, 1,215, 1,114, 1,002, 960, 751 cm^−1^; elemental analysis calcd (%) for C_8_H_7_N_13_O_2_ (317.08): C 30.29, H 2.22, N 57.40; found: C 30.52, H 2.06, N 57.21; IS: 22 J, FS: 350 N.

**Synthesis of ammonium 3-(3-cyano-2-oxido-1,2,5-oxadiazol-4-yl)-1,2,4-triazol-1-ide (5d)**. Ammonia was bubbled through a solution of **1b** (0.88 g, 4.9 mmol) in CH_3_CN (10 ml). The saturated solution was stirred for 0.5 h. The precipitate was filtered off and washed with Et_2_O (2 × 10 mL) to give energetic salt **5d**. Yield 0.83 g (86%); light orange solid; ^1^H NMR (300 MHz, DMSO-*d*_6_): *δ* = 8.37 (s, 1H), 6.35 (br. s, 4H); ^13^C{^1^H} NMR (75.5 MHz, DMSO-*d*_6_): *δ* = 151.0, 150.8, 148.6, 107.8, 98.0; ^14^N NMR (21.7 MHz, DMSO-*d*_6_): *δ* = −361.0 (br. s, NH_4_); IR (KBr): 3,324, 3,146, 2,857, 2,252, 1,621, 1,583, 1,536, 1,453, 1,346, 1,276, 1,249, 1,178, 1,105, 1,057, 970, 874, 832 cm^−1^; elemental analysis calcd (%) for C_5_H_5_N_7_O_2_ (195.05): C 30.78, H 2.58, N 50.24; found: C 30.74, H 2.76, N 49.98; IS: 11 J, FS: 260 N.

**Synthesis of ammonium 5-(2-oxido-4-(1*H*-1,2,4-triazol-3-yl)-1,2,5-oxadiazol-3-yl)tetrazol-1-ide 6**: TMSN_3_ (2.5 mmol, 0.33 ml) and NH_4_F (1 mmol, 0.037 g) were added to a magnetically stirred mixture of cyanofuroxan **3a** (1 mmol) in MeCN (5 ml). The reaction mixture was stirred at 35°C for 10 h until full consumption of substrate **3a** (TLC monitoring). Then, the precipitate formed was filtered off, washed with CH_3_CN (5 ml) and dried in a vacuum desiccator over P_2_O_5_. Yield 0.20 g (84%); beige solid; ^1^H NMR (300 MHz, DMSO-*d*_6_): *δ* = 8.65 (s, 5H); ^13^C{^1^H} NMR (75.5 MHz, DMSO-*d*_6_): *δ* = 150.4, 149.8, 147.7, 147.4, 110.4; ^14^N NMR (21.7 MHz, DMSO-*d*_6_): *δ* = −358.8 (s, NH_4_); ^15^N NMR (60.8 MHz, DMSO-*d*_6_): *δ* = 12.3, −8.0, −9.9, −12.3, −24.7, −58.3, −130.9, −358.1; IR (KBr): 3,342, 3,120, 2,989, 1,598, 1,523, 1,448, 1,416, 1,270, 1,220, 1,145, 1,076, 976, 969, 893, 835 cm^−1^; elemental analysis calcd (%) for C_5_H_6_N_10_O_2_ (238.17): C 25.22, H 2.54, N 58.81; found: C 25.03, H 2.68, N 58.49; IS: 12 J, FS: >360 N.

## 3 Results and discussion

**SCHEME 1:**
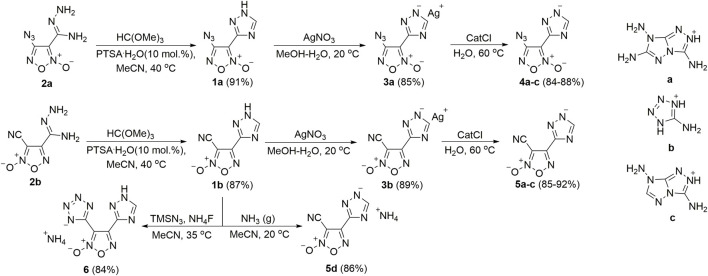
Synthetic route to target energetic salts.

Parent biheterocyclic cores, namely 4-azido-3-(1*H*-1,2,4-triazol-3-yl)furoxan **1a** and 3-cyano-4-(1*H*-1,2,4-triazol-3-yl)furoxan **1b** were synthesized according to our recently reported procedure (Larin et al., 2022) via PTSA-catalyzed cyclocondensation of the available amidrazones **2a,b** with trimethyl orthoformate. Thus formed biheterocyclic compounds **1a,b** were treated with AgNO_3_ to obtain corresponding silver salts **3a,b** which were further subjected to a metathesis reaction with various nitrogen-rich bases (in a form of hydrochlorides) affording two arrays of high-nitrogen energetic salts **4a-c** and **5a-d**. Cyano group in compound **1a** was also converted to the tetrazolate anion through cycloaddition with *in situ* generated TMSN_3_ (Scheme 1). This approach enabled a selective formation of the (tetrazolyl)furoxan ammonium salt **6** bearing neutral 1,2,4-triazole scaffold.

**FIGURE 2:**
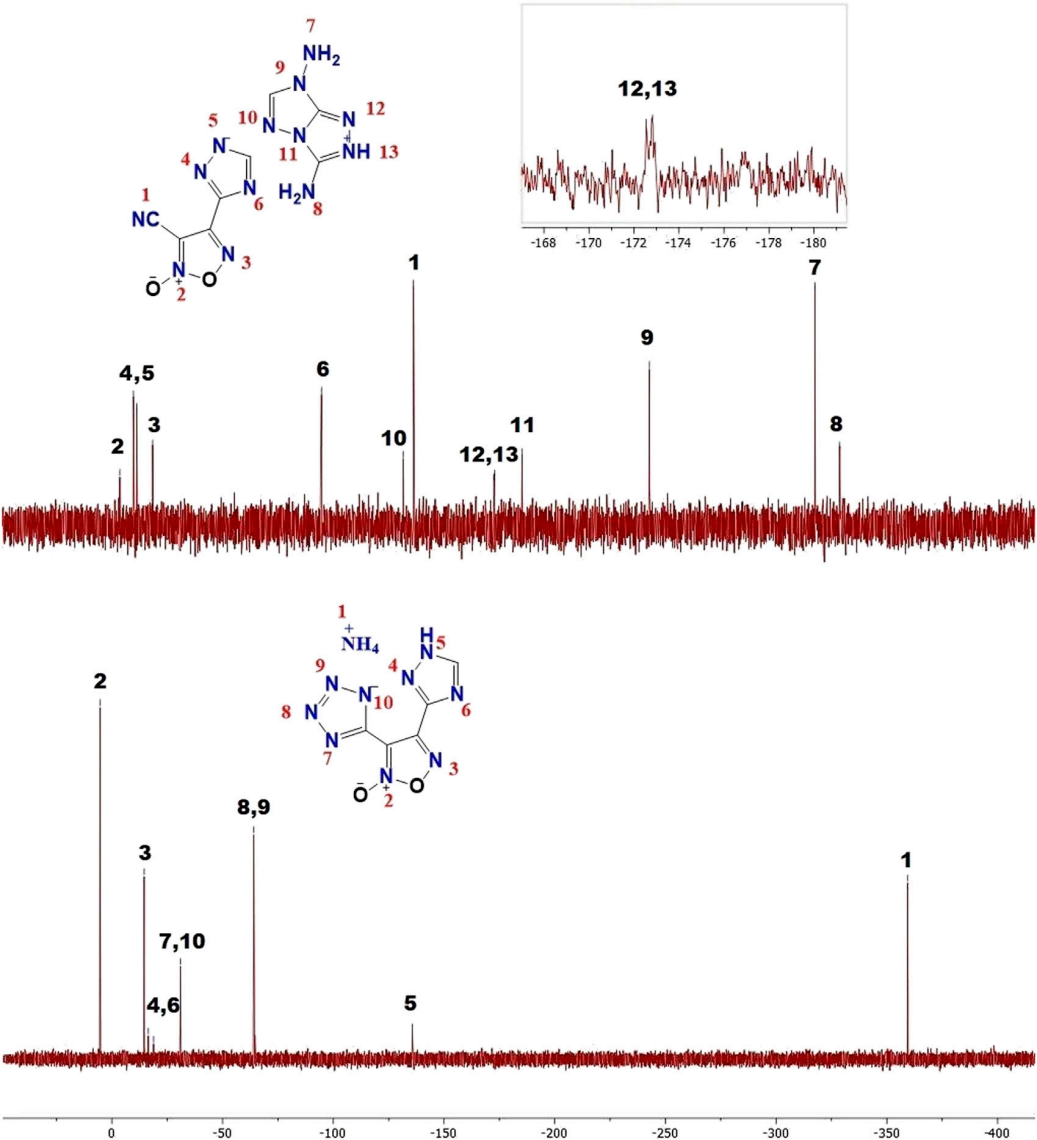
^15^N NMR spectra for compounds **5c** and **6**.

All newly synthesized compounds were fully characterized by IR and multinuclear (^1^H, ^13^C, ^14^N) NMR spectroscopy as well as elemental analysis. Because the synthesized energetic salts have a high nitrogen content, several representatives, namely salts **5c** and **6**, were additionally characterized by ^15^N NMR spectroscopy (Figure 2). The signals were assigned by comparison with similar structures (Thottempudi and Shreeve, 2011; Tang et al., 2021). There are thirteen signals in the ^15^N NMR spectrum of **5c**. The nitrogen atom signals of the two amino groups are observed in the upfield region (δ = −317.5 ppm (N7) and δ = −328.7 ppm (N8). The resonance peaks for N12 and N13 in the cation counter-part are nearly overlapping for structure **5c**. The 1,2,4-triazolium anion has characteristic signals at δ = −9.6 (N4), δ = −11.2 (N5), δ = −94.6 (N6), while corresponding peaks of the furoxan ring are located at δ = 3.6 (N2) δ = −18.4 ppm (N3). Only eight signals are presented in the ^15^N NMR spectrum of **6**. The chemical shift of the nitrogen atom of the ammonium cation (N1) is found at δ = −358.1 ppm which correlates with the corresponding ^14^N NMR spectrum of **6**. Due to the symmetric structure of the tetrazolate anion in **6**, two signals were found at δ = −24.7 ppm (N7, N10) and −58.3 ppm (N8, N9). Two signals of nitrogen atoms of the furoxan ring were observed in a downfield region at δ = 12.3 ppm (N2) and −8.0 ppm (N3).

**FIGURE 3:**
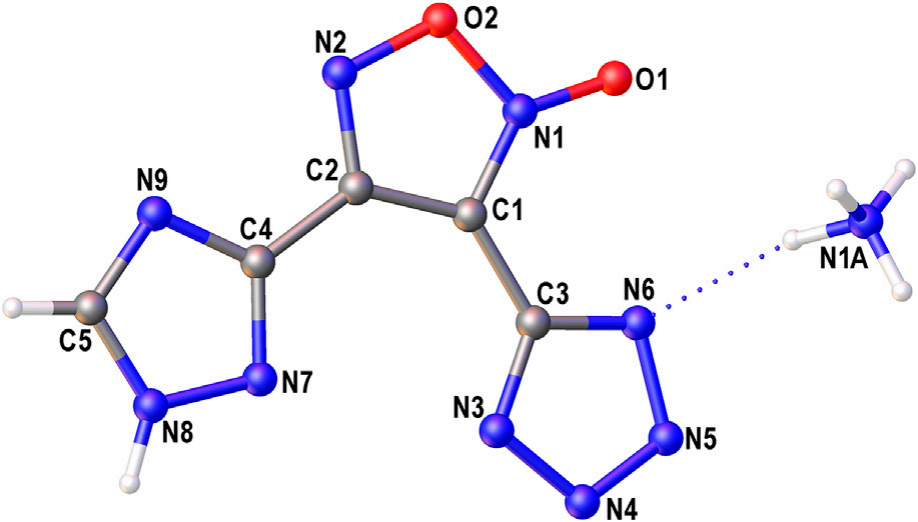
The general view of symmetry independent part of compound **6**. Non-hydrogen atoms are drawn by probability ellipsoids of atomic displacements (*p* = 0.5).

**FIGURE 4:**
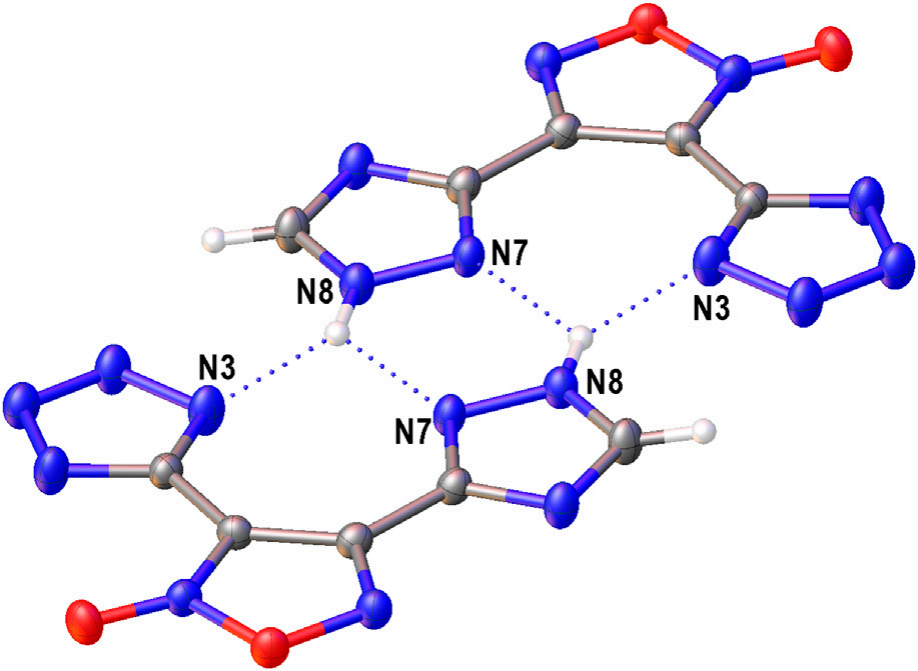
The centrosymmetric dimers of the anions in crystal of **6** stabilized by bifurcate H-bonds (dotted lines).

**FIGURE 5:**
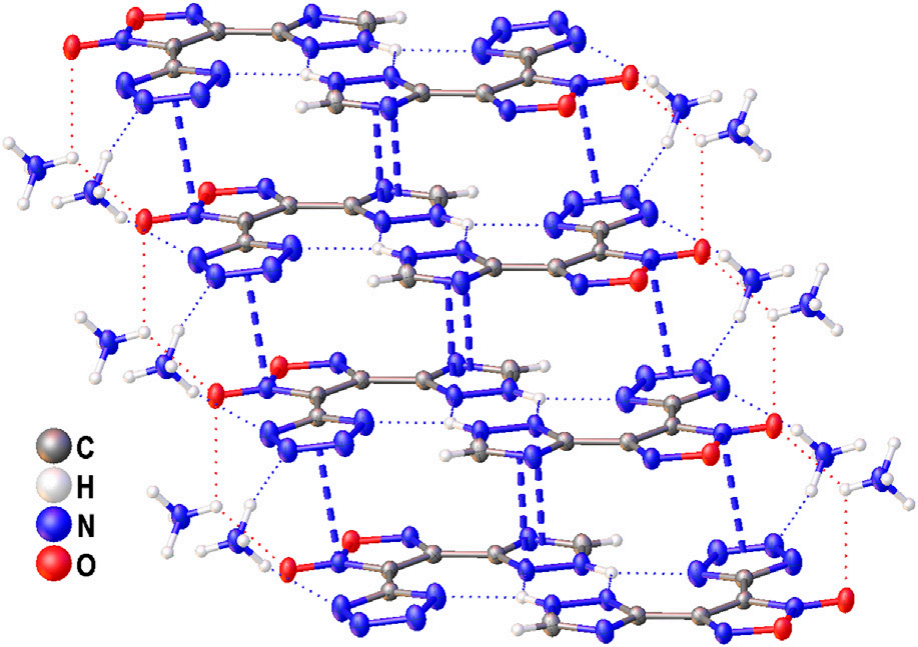
A fragment of infinite column in crystal of **6** stabilized by cation-anion H-bonds (dotted lines) and stacking interactions between anions (dashed lines).

**FIGURE 6:**
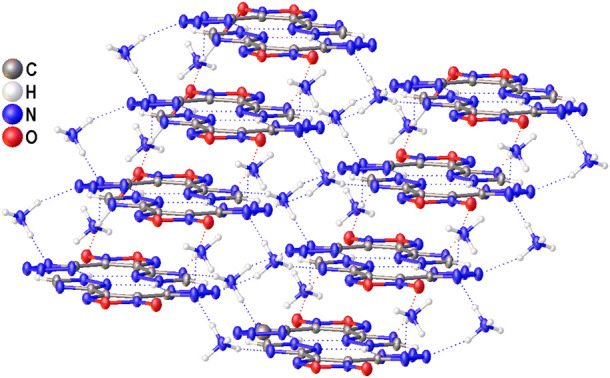
A fragment of crystal packing of **6** demonstrating the aggregation of infinite columns.

The structure of compound **6** was further confirmed by X-ray diffraction study (Figure 3). In crystal, the anionic moiety is nearly planar: the C1C2C4N7 and C2C1C3N3 torsion angles are 1.95° and 6.36°, respectively, while the mean deviation from plane composed by non-hydrogen atoms is only 0.0436 Å. The main packing motif for **6** is the aggregation of anions into centrosymmetric dimers (Figure 4) by means of two bifurcate H-bonds between the N8(H) groups of triazolyl cycle and the N3 and N7 nitrogen atoms of tetrazole and triazolyl, respectively (N8…N3 and N8…N7 distances are 2.811 and 2.923 Å, correspondingly). The further binding of these dimers is determined by the ammonia cations (Supplementary Figure S1) which form 1) one weak bifurcate NH…O H-bond with exocyclic furoxan oxygen atoms (N…O 3.018 and 3.051 Å), 2) two moderate NH…N hydrogen bonds with the tetrazole fragments (N…N 2.876, 2.954 Å, ∠(NHN) 160.6–161.2°) and 3) one moderate NH…N H-bond with the triazole ring (N…N 2.936 Å, ∠(NHN) 167.2°). The two former types of H-bonds aggregate the nearly planar dianionic dimers into infinite columns which are additionally stabilized by furoxan…tetrazole and triazole…triazole stacking interactions (the interplane distance is 3.140 Å, Figure 5). Despite the whole crystal packing pattern resembles the layer-like supramolecular aggregation, the mentioned columns are shifted against each other due to the mentioned NH…N H-bonds between the cation and triazole ring (Figure 6).

The compound **6** was found to be an excellent example of the influence of supramolecular forces on the molecular structure. Although the planar conformation of the anion is expected for this class of compounds according to the processing of Cambridge Structural Database (Groom et al., 2016), the DFT calculations of several isolated models demonstrate quite the opposite picture (Supplementary Figures S2–S8). For instance, the triazolyl cycle is perpendicular to the tetrazolyl-furoxan plane in the isolated anion even if the non-specific solvation effects are accounted for. The significant violation of π-conjugation is also found for the isolated clusters which were optimized using different fragments of crystal structure of **6** as starting geometries. Here, two factors should be noted: the interplay between triazole and tetrazole moieties to form π-conjugation with the central furoxan ring and the crystal media influence (see Supplementary Table S1 for the information on torsion angles). As an example, the SCRF calculations of isolated anion resulted in the increase of triazole-furoxan conjugation and the decrease of the tetrazole-furoxan one. In its turn, only the modelling of a cluster containing two anionic dimers stabilized by stacking interactions between them and NH…N H-bonds with four cations (see the discussion of crystal packing above) produced the conformation with rather small values of C1C2C4N7 and C2C1C3N3 torsion angles (19.4° and 12.9°, respectively). Note that the only one of the protonated isomers of **6** is totally planar (torsion angles less than 1°) that is rationalized by the presence of strong intramolecular N3-H…N7 hydrogen bond in this case (N3…N7 2.758 Å, Supplementary Figure S5).

The combination of mentioned strong interionic interactions in crystal of **6** together with its column-type crystal packing and non-favorable planar conformation of the anion may be the reason of moderate physicochemical properties of **6** (see below). Indeed, HEDMs with the layer packing are usually characterized by rather high impact sensitivity (IS) values (Tian et al., 2018), possibly due to the asymmetry of tensions in the equilibrium structure which should be worked upon impact. A similar behavior can be supposed for compounds with stressed conformations such as **6**. At the same time, the formation of strong directional H-bonds can also decrease the density of a crystal. Keeping all this in mind, it is still interesting to compare structure-property relationships for **6** and two similar compounds—azidotetrazolylfuroxan (CSD refcode DUQGUT) (Fershtat et al., 2015a) and nitrotetrazolylfuroxan (CSD refcode TILMEI) (Liang et al., 2013). The TILMEI and DUQGUT structures have been already analyzed by us when the role of cation in physico-chemical properties of tetrazolyl-furoxan salts was under consideration (Larin et al., 2019). The comparison of these two structures with **6** is vital to determine the role of a furoxan’s substituent in these salts.

Despite similar composition of **6**, TILMEI and DUQGUT, the IS value for **6** (12 J, see Table 1 below) is pronouncedly larger than that for the nitro and azido salts (2.0 and 2.2 J, respectively) (Liang et al., 2013; Larin et al., 2019). As mentioned above, this can be rationalized by the combination of factors. First of all, the crystal packings of TILMEI and DUQGUT structures are both more isotropic than the column-type packing of **6** (see Supplementary Figure S9). Furthermore, the crystal geometries of azidotetrazolylfuroxan and nitrotetrazolylfuroxan anions are rather close to those in the equilibrium isolated structures: the rmsd value for non-hydrogen atoms are 0.0703 and 0.2628 Å for DUQGUT and TILMEI (Supplementary Figure S10), whereas the energy difference between the isolated and crystal (optimized hydrogen atoms) geometries is 3.2 and 4.3 kcal mol^−1^, respectively. The crystal structure of anion in **6** is significantly more stressed: the corresponding values are larger due to the mentioned rotation of triazole cycle (0.7295 Å and 8.1 kcal mol^−1^). Finally, the low IS values for DUQGUT and TILMEI agree well with the presence of explosophoric N_3_ group in the former and the dense packing of the latter (1.85 vs. 1.71 g cm^−3^ for **6**).

**TABLE 1:** Physical properties and detonation parameters.

Compound	*T*_d_ ^a^ [°C]	*ρ* ^b^ [g cm^−3^]	*N* ^c^ [%]	[N + O]^d^ [%]	Δ*H* ^0^_f,solid_ ^e^ [kJ mol^−1^]	Δ*H* ^0^_f,solid_ [kJ g^−1^]	*D* ^f^ [km s^−1^]	*P* ^g^ [GPa]	*IS* ^h^ [J]	*FS* ^i^ [N]
**1a**	154	1.70	57.7	74.2	623	3.2	8.0	29	4	270
**4a**	155	1.68	64.4	73.6	1,044	3.0	7.9	27	8	>360
**4b**	150	1.80	65.2	76.7	896	3.2	8.4	32	9	>360
**4c**	152	1.68	63.1	72.7	1,095	3.3	7.9	28	5	260
**1b**	229	1.55	47.2	65.2	453	2.5	7.0	22	19	220
**5a**	153	1.68	59.0	68.7	834	2.5	7.5	25	14	290
**5b**	172	1.76	58.6	70.7	703	2.7	7.9	29	21	250
**5c**	154	1.66	57.4	67.5	887	2.8	7.5	26	22	350
**5d**	232	1.61	50.3	66.7	344	1.8	7.2	23	11	260
**6**	217	1.64	58.8	72.2	467	2.0	7.5	24	12	>360
**TNT**	275	1.64	18.5	60.8	−62	−0.3	6.9	23	30	>360
**PETN**	165	1.78	17.7	78.5	−561	−1.8	8.4	32	3	70

a Decomposition temperature (DSC, 5 K min^−1^).

b Density (gas pycnometer, 298 K).

c Nitrogen content.

d Nitrogen and oxygen content.

e Calculated enthalpy of formation.

f Calculated detonation velocity.

g Calculated detonation pressure.

h Impact sensitivity.

i Friction sensitivity.

It is interesting to note that the crystal density value does not correlate with the impact sensitivity for **6** and DUQGUT: crystals of azidotetrazolylfuroxan are slightly less dense (1.71 g cm^−3^). The Δ_OED_ formalism (Dalinger et al., 2018a; Dalinger et al., 2018b) was utilized to explore the influence of a substituent in tetrazolylfuroxan salts on density of their crystals. This approach allows to estimate densification (Δ_OED_) of a system upon the gas-to-crystal transition as it is based on the comparison of volumes which are filled by a system in the isolated state (0.0004 a. u. isosurface of electron density function) and in crystal. More information can be gathered by means of the exhaustive partitioning of real 3D space (for example, by means of the “Atoms in Molecules” theory) that allows to decompose the crystal density into the density of chemically meaningful fragments. This technique was found to be particularly helpful for the analysis of salts and co-crystals (Larin et al., 2019). The details of such calculations for **6**, DUQGUT and TILMEI are listed in Supplementary Table S2 of Supporting Information.

It was found that the presence of substituent with the increasing ability to be densified upon crystal formation positively affects the densification of the whole salt. In the isolated states, the triazole ring in **6** is less dense that the azide moiety in DUQGUT while it becomes considerably denser than N_3_ in corresponding crystals. The large densification of triazole evidently produced by a greater number of short non-covalent interactions (rather strong H-bonds in the case of **6**) increases the densification of all other fragments. As a result, the structure of **6** is slightly denser than that of DUQGUT. In the same manner, the dense NO_2_ group usually decreases its volume to a large extent due to the participation in a number of shortened intermolecular contacts; the presence of this group in TILMEI leads to a pronounced densification of this salt and its large crystal density. Finally, it is interesting to note, that the statistical consideration of Δ_OED_ values for fragments of **6**, DUQGUT and TILMEI also indicates the influence of crystal packing effects on the impact sensitivity. The sample variance is the same in TILMEI and DUQGUT (0.007 g^2^ cm^−6^) while it is larger for **6** (0.010 g^2^ cm^−6^). Indeed, a larger variance corresponds to a more dispersive densification of fragments that is obtained by less isotropic distribution of non-covalent interactions.

**FIGURE 7:**
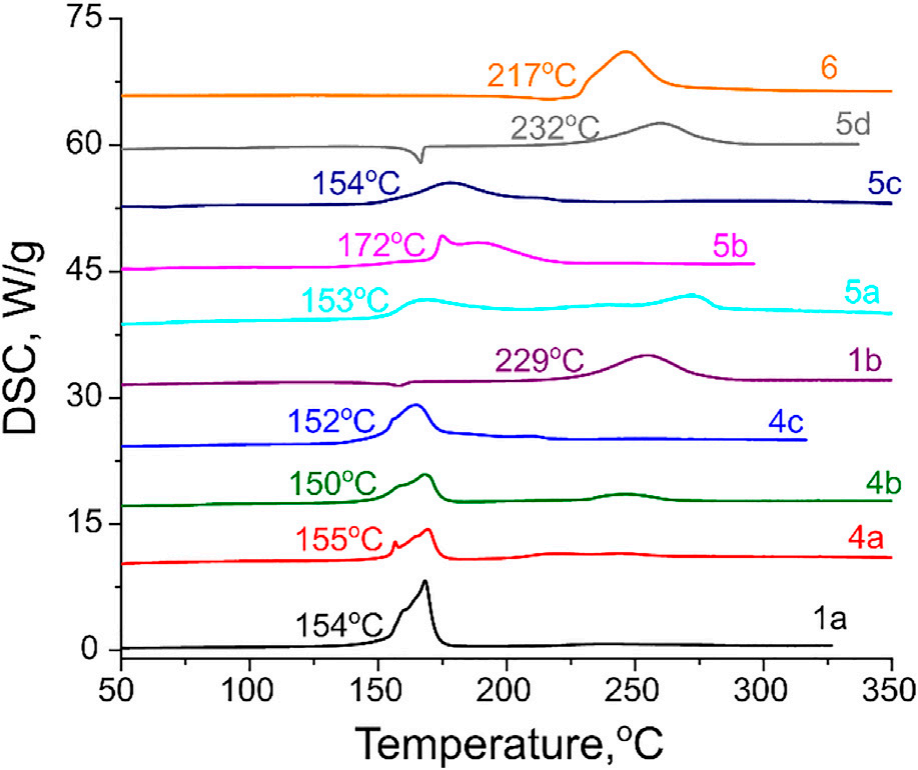
DSC traces for synthesized energetic compounds taken at 5 K min^−1^ heating rate. The extrapolated onsets of exothermic decomposition peaks are indicated.

Physicochemical and sensitivity properties as well as detonation performance of all synthesized compounds were evaluated and the results are summarized in Table 1. As for thermal stability, most of the investigated salts have acceptable thermal decomposition onsets in the range of 150–172 ^о^С. The exceptions are neutral compound **1b** and two ammonium salts **5d** and **6**, which all have thermal stability in conventional linear heating DSC tests above 200°C (Figure 7).

Synthesized compounds have good densities (1.61–1.80 g cm^−3^) and considerable amount of nitrogen and oxygen within the molecule (65%–74%). Calculated enthalpies of formation of the synthesized energetic salts are in a range of 344–1,095 kJ mol^−1^. Based on the experimental densities and calculated enthalpies of formation, detonation performance of all synthesized energetic salts was estimated and compared to benchmark explosives TNT and PETN (all calculations were performed using PILEM software). Detonation velocity values were found to be within 7.0–8.4 km s^−1^, while the calculated detonation pressures fall in a range of 22–32 GPa. Therefore, the energetic performance of these new compounds outperform benchmark explosive trinitrotoluene (TNT), but are inferior to booster explosive pentaerythritol tetranitrate (PETN). These two contemporary energetic materials also serve as limits for synthesized compounds from the mechanical sensitivity aspect. Impact sensitivity for (1,2,4-triazolyl)furoxan energetic salts varies from sensitive 5 J for **4c** to less sensitive **5b**,**c** with 21–22 J. Analyzed species are not very sensitive to friction stimulus, e.g., compounds **4a**,**b**, **6** are even not sensitive at all.

## 4 Conclusion

In conclusion, an array of novel nitrogen-rich energetic salts comprising of the (1,2,4-triazolyl) furoxan core was designed and synthesized. All target high-energy materials were prepared in high yields starting from readily available furoxan precursors bearing amidrazone functionality and well characterized with IR and multinuclear NMR spectroscopy, elemental analysis and X-ray diffraction study. According to the X-ray diffraction study, the large densification of the 1,2,4-triazole heterocycle evidently produced by a greater number of short non-covalent interactions (rather strong H-bonds in the case of **6**) increases the densification of all other fragments. Thermal stabilities of the synthesized energetic materials were determined using differential scanning calorimetry. It was found that most of the investigated salts have acceptable thermal decomposition onsets in the range of 150°С–172°С, while two ammonium salts **5d** and **6** possess excellent thermal stabilities far above 200°C. A combination of good experimental densities (1.61–1.80 g cm^−3^) and high positive calculated enthalpies of formation (344–1,095 kJ mol^−1^) provides good detonation performance of the synthesized energetic salts (*D* = 7.0–8.4 km s^−1^; *p* = 22–32 GPa) exceeding that of benchmark explosive TNT (*D* = 6.9 km s^−1^; *p* = 23 GPa) and reaching the limits of booster PETN (*D* = 8.4 km s^−1^; *p* = 32 GPa). At the same time, sensitivities of the prepared (1,2,4-triazolyl) furoxan salts to impact are quite moderate, while their sensitivities to friction are even lower, mainly on the level of insensitive TNT. To the best of our knowledge, the present study is one of the rarely reported high-energy materials incorporating a 1,2,4-triazole heterocycle as an anion counter-part and a first example of (1,2,4-triazolyl) furoxan energetic salts.

## Data Availability

The datasets presented in this study can be found in online repositories. The names of the repository/repositories and accession numbers can be found in the article/Supplementary Material.
