# Supplementary material for: Novel family of nitrogen-rich energetic (1,2,4-triazolyl) furoxan salts with balanced performance
Source: Front Chem. 2022 Sep 12;10:1012605. doi: 10.3389/fchem.2022.1012605 (PMC9510683; doi:10.3389/fchem.2022.1012605)
Supplement: Supplementary file 1 [file DataSheet1.PDF]

Supporting Information for

Novel Family of Nitrogen-Rich Energetic (1,2,4-Triazolyl)furoxan Salts with Balanced Performance

Alexander A. Larin[1,2], Alla N. Pivkina[3], Ivan V. Ananyev[4,5], Dmitry V. Khakimov[1], Leonid L. Fershtat[1*]

[1]N.D. Zelinsky Institute of Chemistry, Russian Academy of Sciences, Leninsky prosp., 47, 119991 Moscow, Russian Federation

[2]National Research University Higher School of Economics, Myasnitskaya str., 20, 101000 Moscow, Russian Federation

[3]N.N. Semenov Federal Research Centre for Chemical Physics, Russian Academy of Sciences, Kosygin Str., 4, 119991 Moscow, Russian Federation

[4]N.S. Kurnakov Institute of General and Inorganic Chemistry, Russian Academy of Sciences, GSP-1, Leninsky prosp., 31, 119991 Moscow, Russian Federation

[5]A.N. Nesmeyanov Institute of Organoelement Compounds, Russian Academy of Sciences, Vavilova str., 28, 119991 Moscow, Russian Federation

*** Correspondence:**
Leonid L. Fershtat
fershtat@bk.ru

Table of Contents

S1. Crystallographic details 2

S2. Copies of NMR spectra 7

S1. Crystallographic details

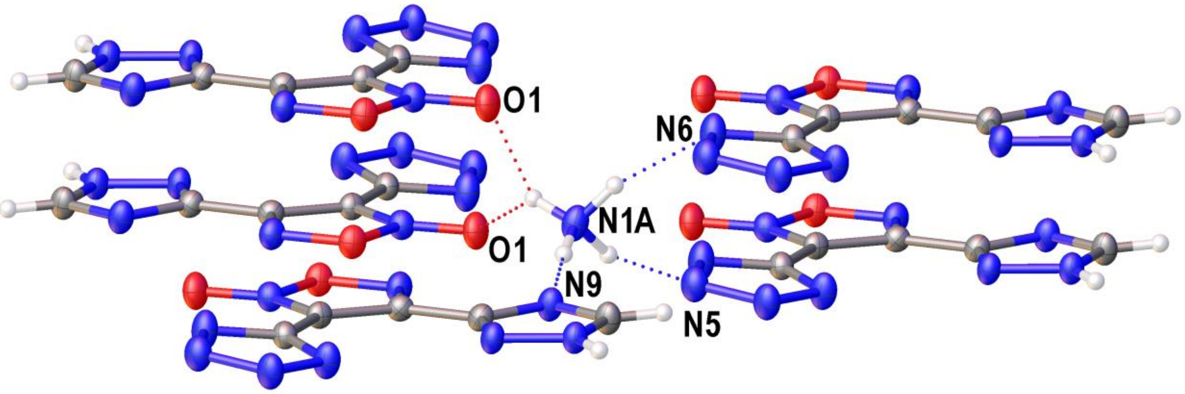

Fig. S1. The H-bonds formed by the ammonia cation in crystal of **6**.

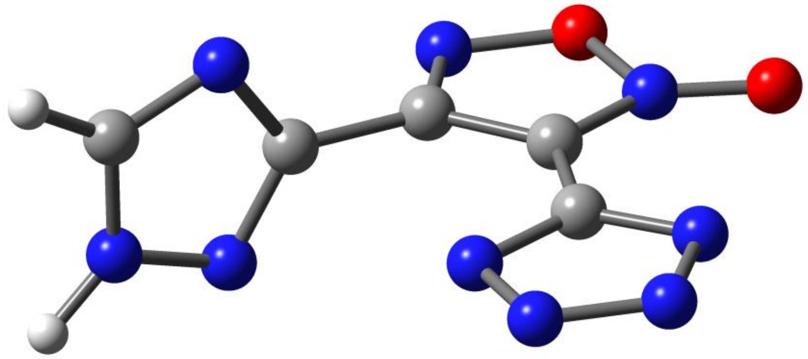

Fig. S2. The DFT optimized structure of the isolated anion of **6** (without solvation model).

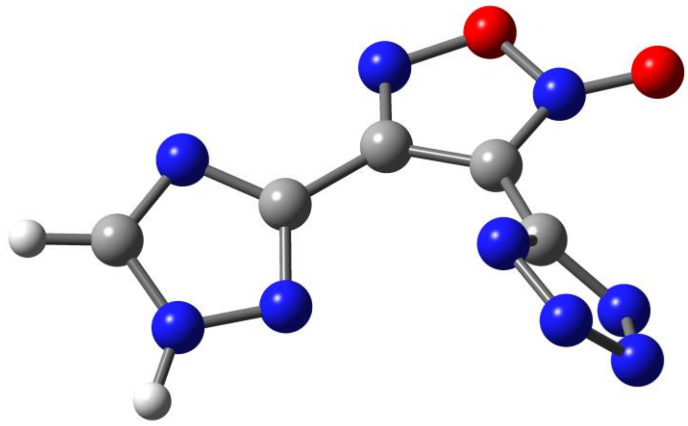

Fig. S3. The DFT optimized structure of the isolated anion of **6** (SCRF, ε=72).

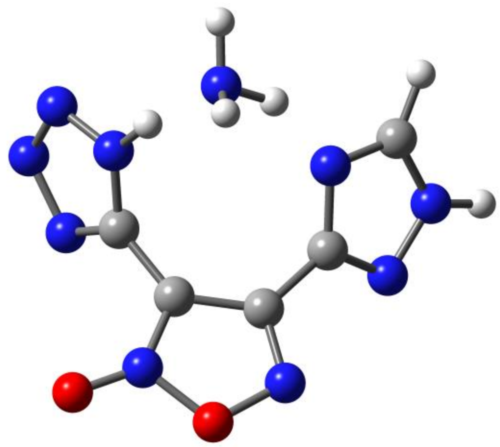

Fig. S4. The DFT optimized structure of the closest ionic pair from crystal of **6** (SCRF, ε=72).

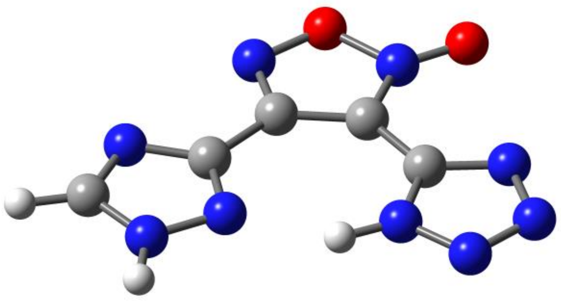

Fig. S5. The DFT optimized structure of the protonated anion of **6** (the H-bonded isomer).

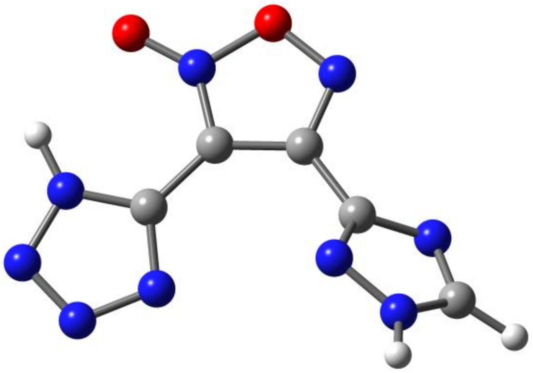

Fig. S6. The DFT optimized structure of the protonated anion of **6** (the isomer without NH…N H-bond).

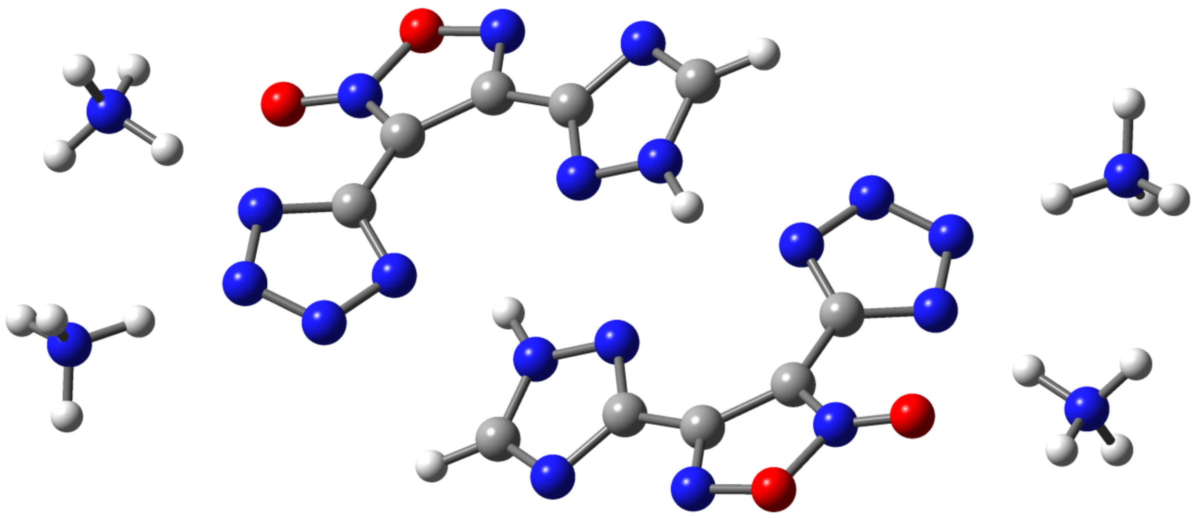

Fig. S7. The DFT optimized structure of a cluster of **6** having four cations and two anions (centrosymmetric dimer).

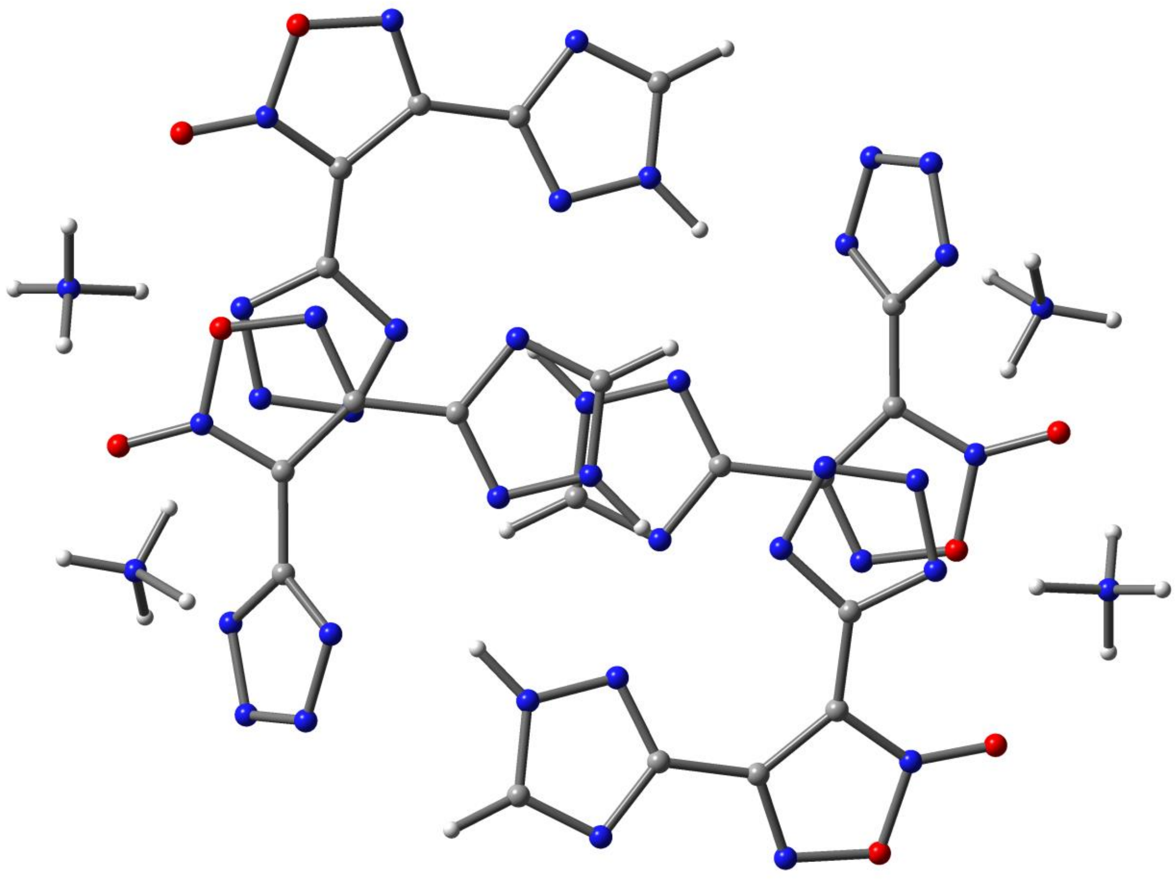

Fig. S8. The DFT optimized structure of a cluster of **6** having four cations and four anions (a fragment of infinite column).

Table S1. The values of C1C2C4N7 and C2C1C3N3 torsion angles (°) in the calculated structures modelling the anion of **6**.

Model	C1C2C4N7	C2C1C3N3	Sum of angles
Isolated anion	85.4	2.0	87.4
Isolated anion with SCRF	23.6	53.0	76.6
Closest ionic pair	47.5	71.1	118.6
Protonated anion (H-bonded isomer)	0.04	0.06	0.1
Protonated anion (isomer wo H-bond)	44.2	4.8	49.0
4 cations and 2 anions	16.7	29.0	45.7
4 cations and 4 anions (A)	19.4	12.9	32.3
4 cations and 4 anions (B)	4.2	43.2	47.4

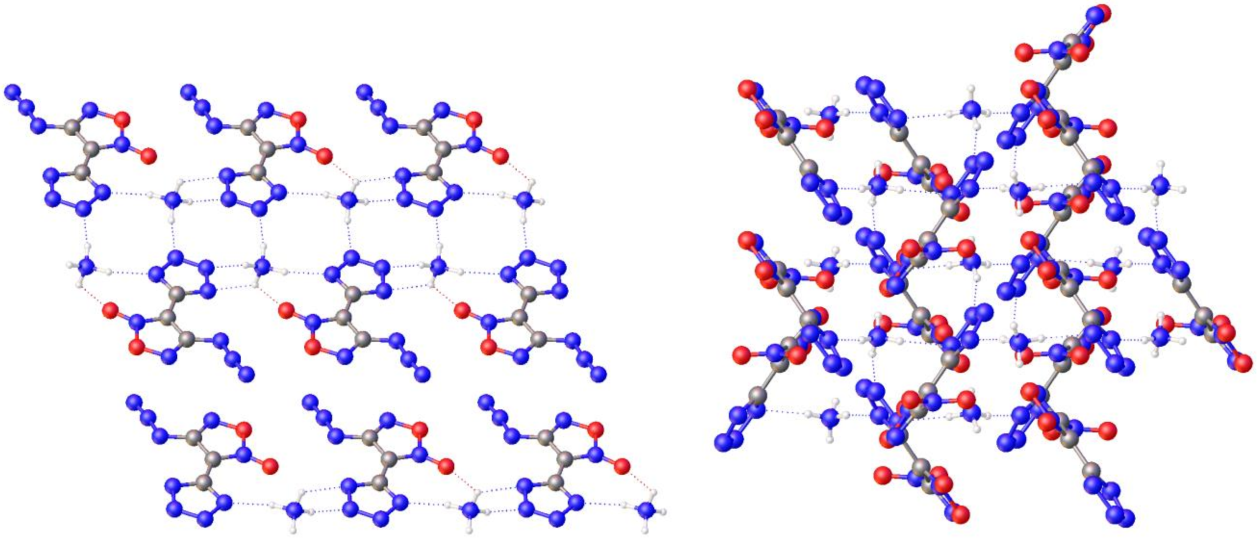

Fig. S9. Fragments of crystal packing of the DUQGUT (left) and TILMEI (right) structures.

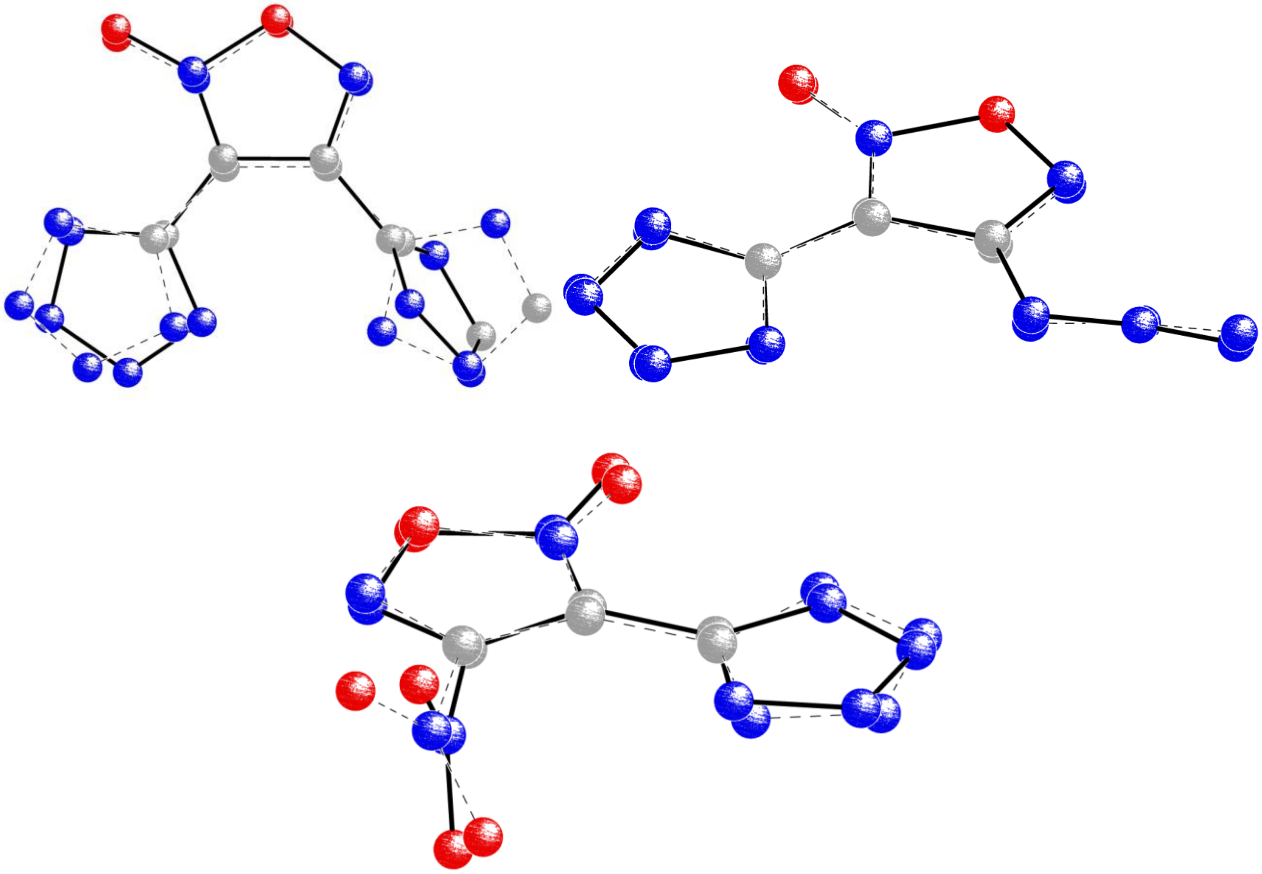

Fig. S10. The best root-mean-square overlap of non-hydrogen atoms for the DFT optimized isolated anions (full lines) and their crystal structures (by dashed lines) in **6** (top left), DUQGUT (top right) and TILMEI (bottom).

Table S2. The Δ_{OED} parameters for **6**, TILMEI and DUQGUT.

Crystal	**6**			TILMEI			DUQGUT		
R=	Triazole			Azide			Nitro		
	di_{sol}	d_{cryst}	Δ_{OED}	di_{sol}	d_{cryst}	Δ_{OED}	di_{sol}	d_{cryst}	Δ_{OED}
Furoxan	1.558	2.075	0.517	1.589	2.086	0.497	1.569	2.020	0.451
Tetrazole	1.263	1.739	0.476	1.254	1.801	0.547	1.399	1.784	0.386
R	1.225	1.612	0.387	1.448	2.020	0.572	1.268	1.544	0.277
Ammonia	0.758	1.054	0.296	0.758	1.137	0.379	0.758	1.035	0.277
Whole (based on the independent pair)	1.273	1.714<br>*1.714*[a]	0.441	1.328	1.852<br>*1.852*[a]	0.524	1.277	1.706<br>*1.706*[a]	0.428

[a] the crystal density values from the X-ray diffraction data.

S2. Copies of NMR spectra

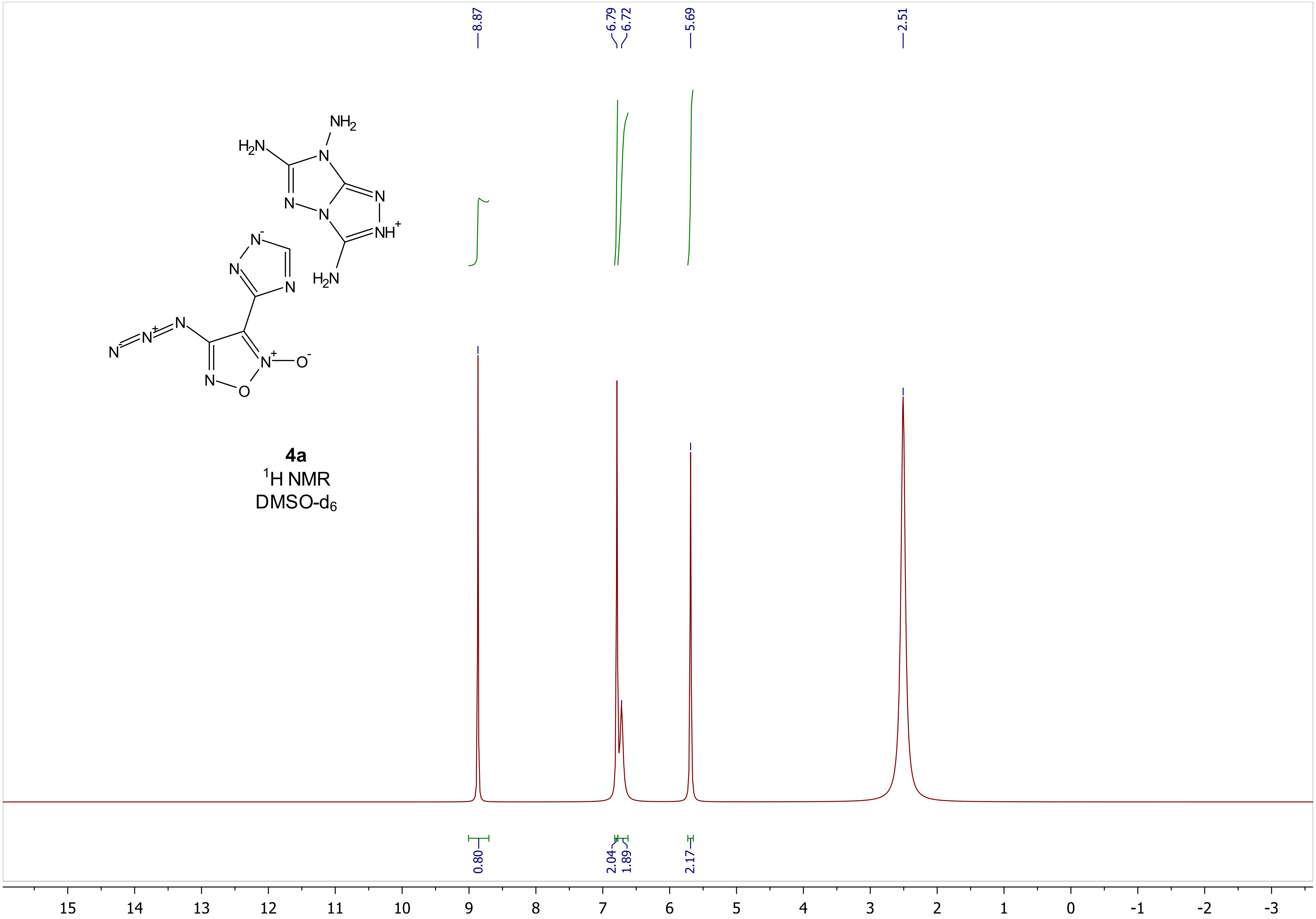

4a
^{1}H NMR
DMSO-d_6
8.87
6.79
6.72
5.69
2.51
0.80
2.04
1.89
2.17
15 14 13 12 11 10 9 8 7 6 5 4 3 2 1 0 -1 -2 -3

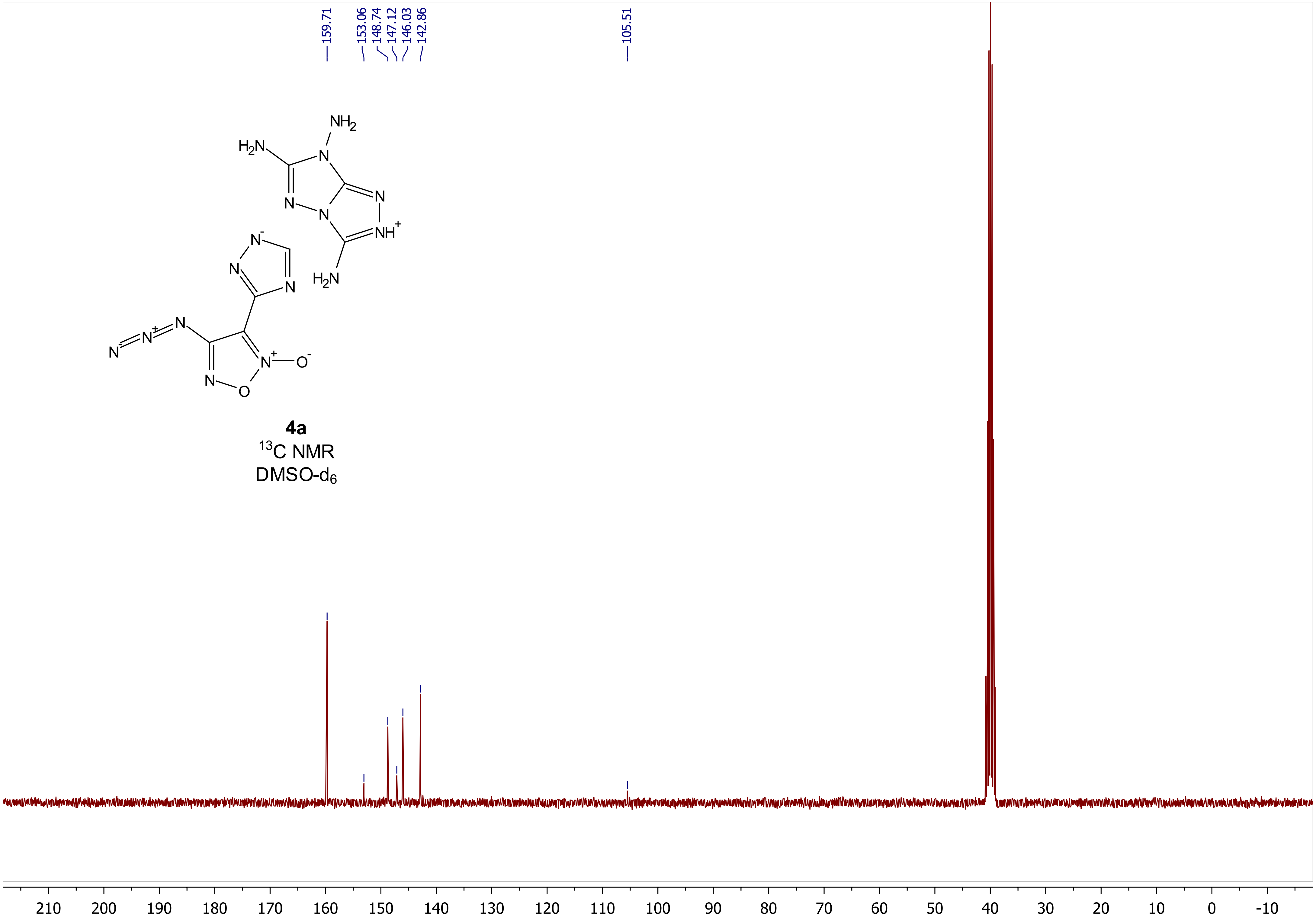

4a

^{13}C NMR

DMSO-d_6

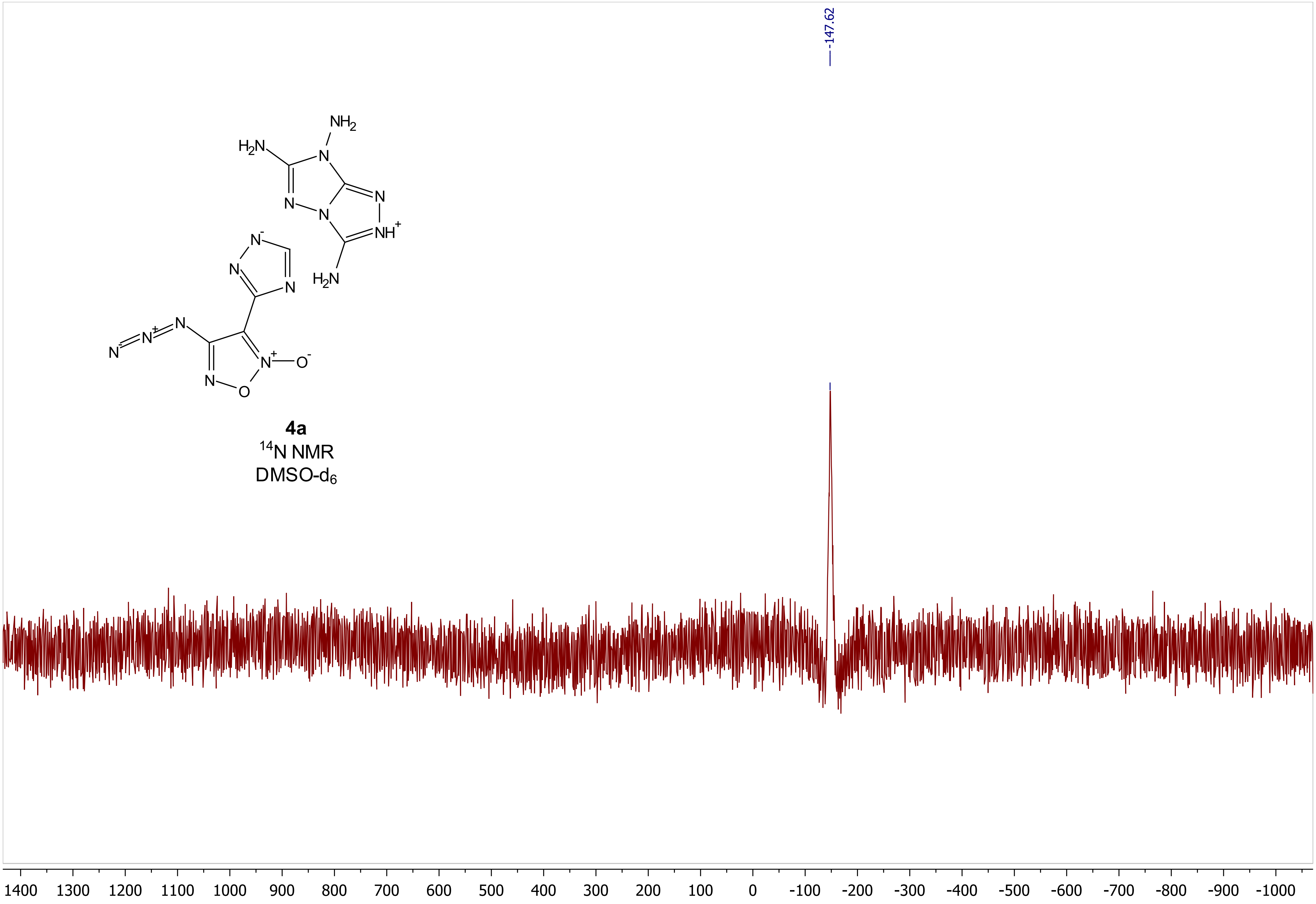
-147.62
4a
14N NMR
DMSO-d6
1400 1300 1200 1100 1000 900 800 700 600 500 400 300 200 100 0 -100 -200 -300 -400 -500 -600 -700 -800 -900 -1000

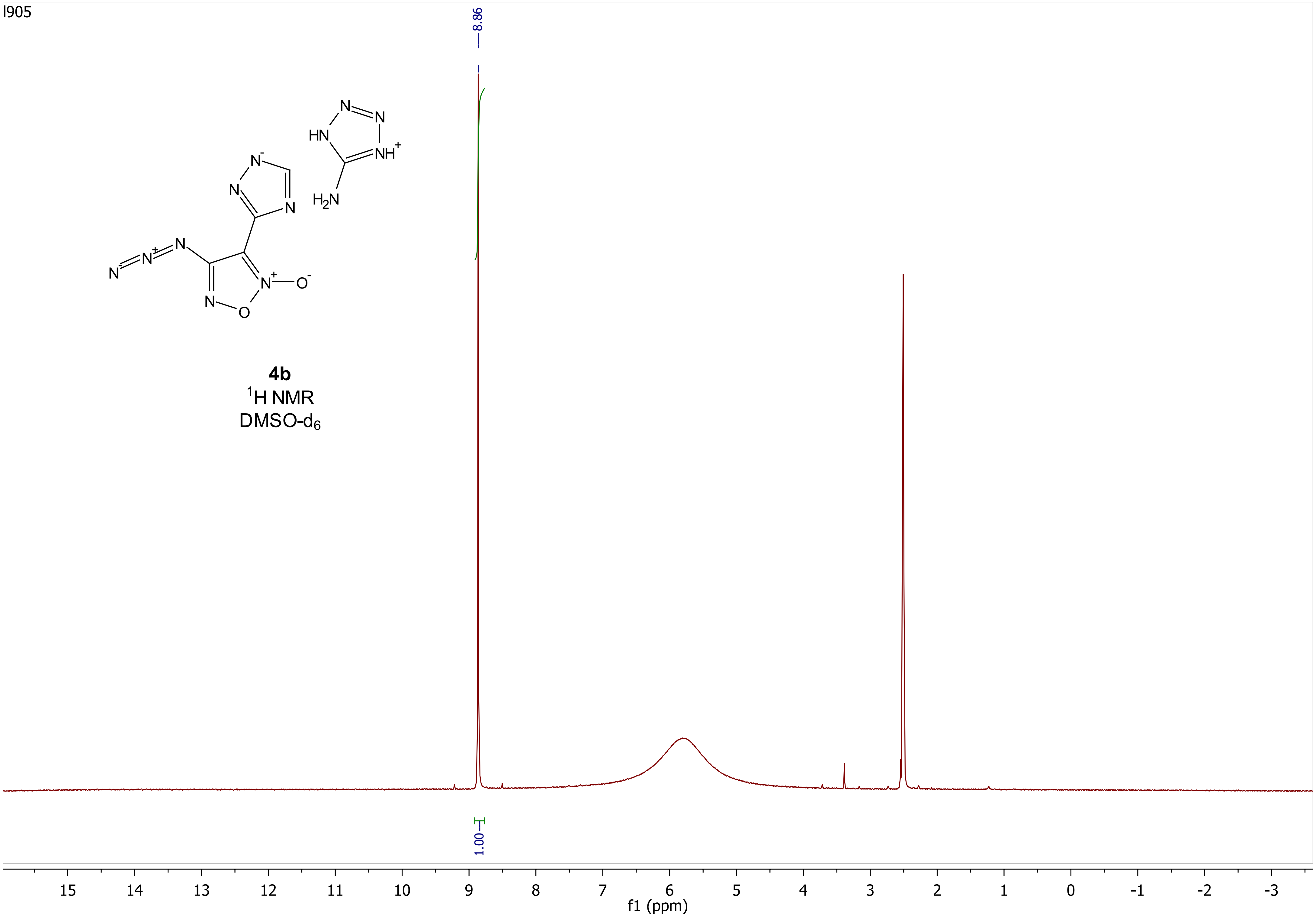
I905
8.86
4b
^{1}H NMR
DMSO-d_6
1.00
15 14 13 12 11 10 9 8 7 6 5 4 3 2 1 0 -1 -2 -3
f1 (ppm)

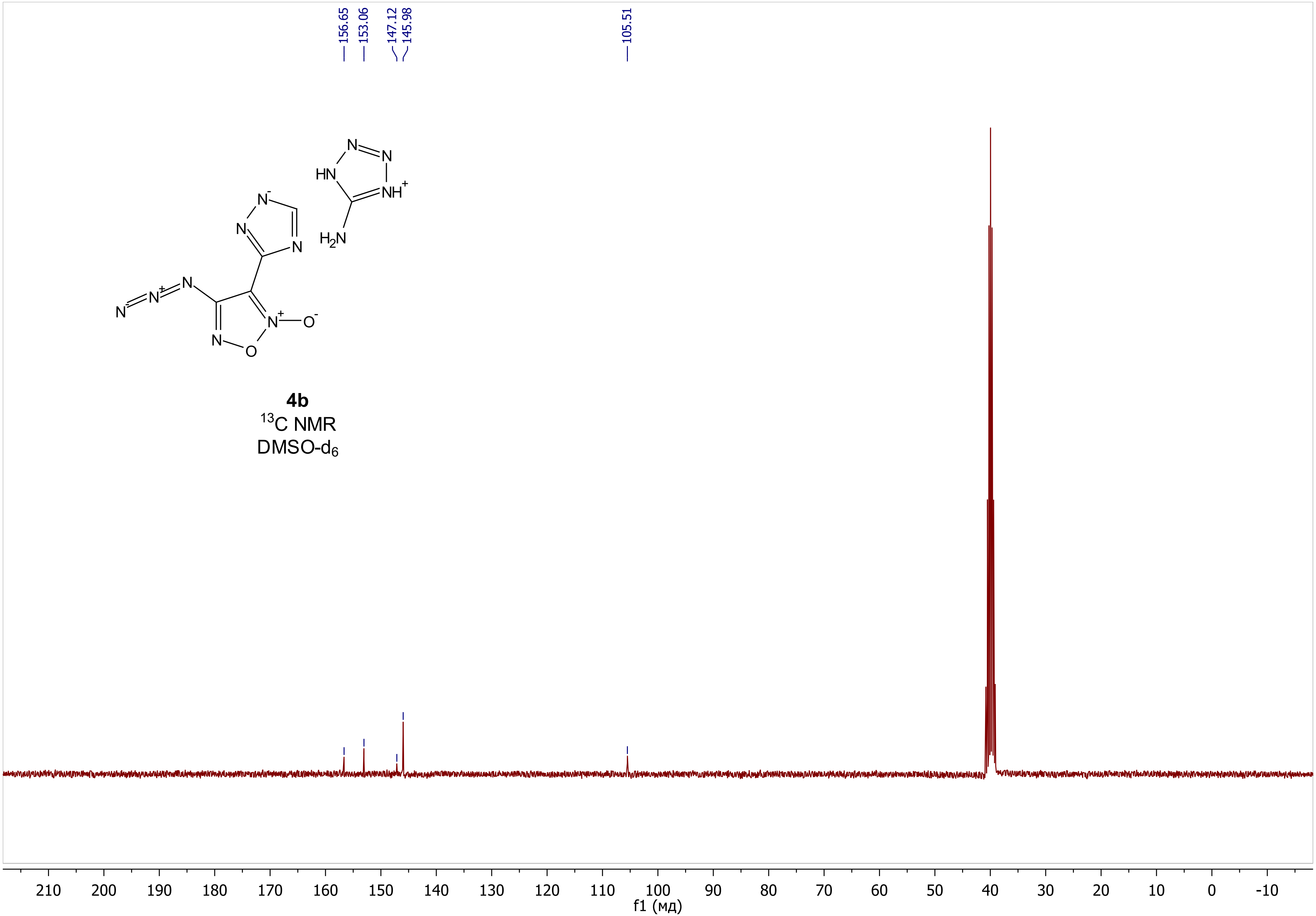

156.65
153.06
147.12
145.98
105.51
4b
13C NMR
DMSO-d6
210 200 190 180 170 160 150 140 130 120 110 100 90 80 70 60 50 40 30 20 10 0 -10
f1 (мд)

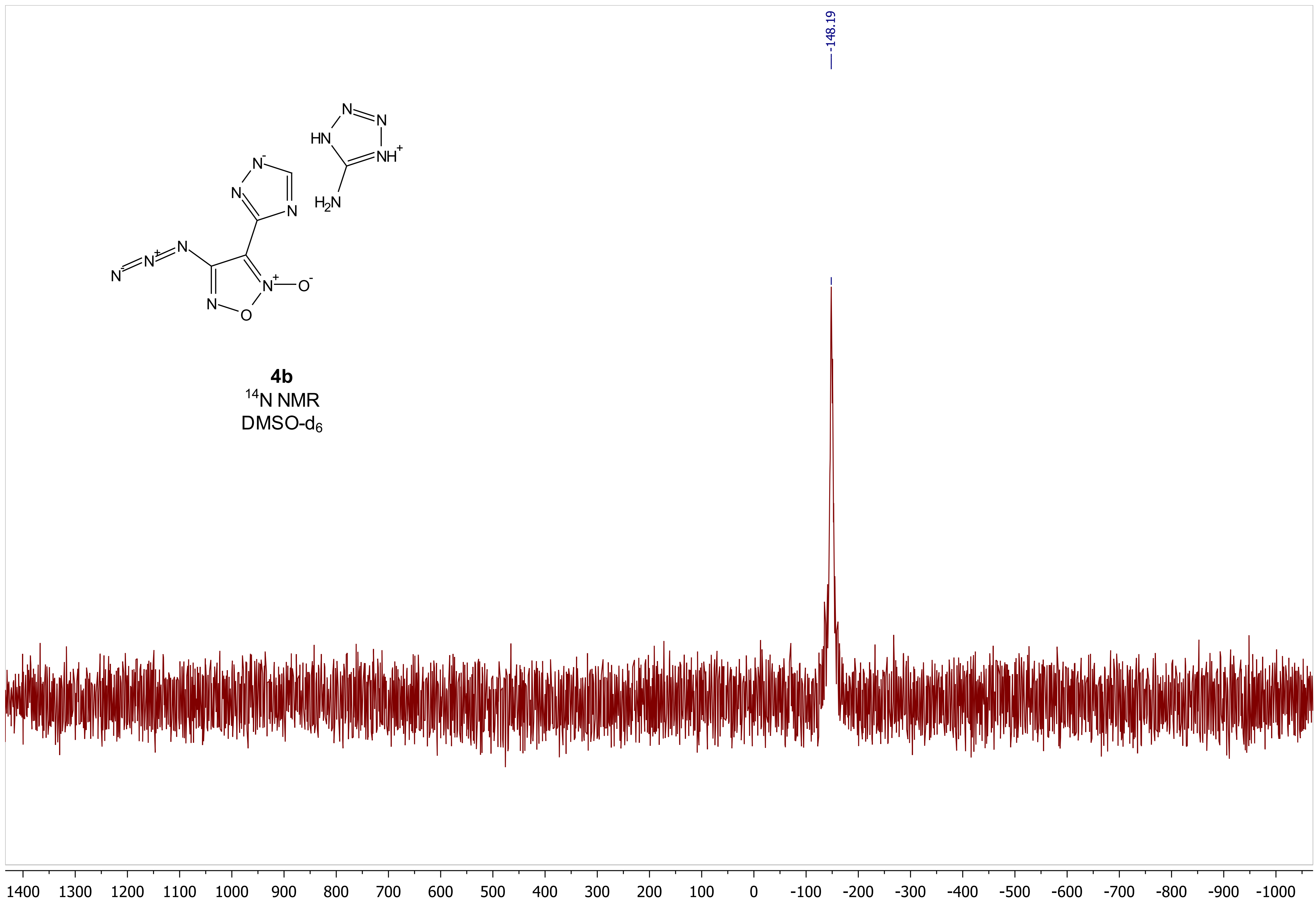
-148.19
4b
14N NMR
DMSO-d6
1400 1300 1200 1100 1000 900 800 700 600 500 400 300 200 100 0 -100 -200 -300 -400 -500 -600 -700 -800 -900 -1000

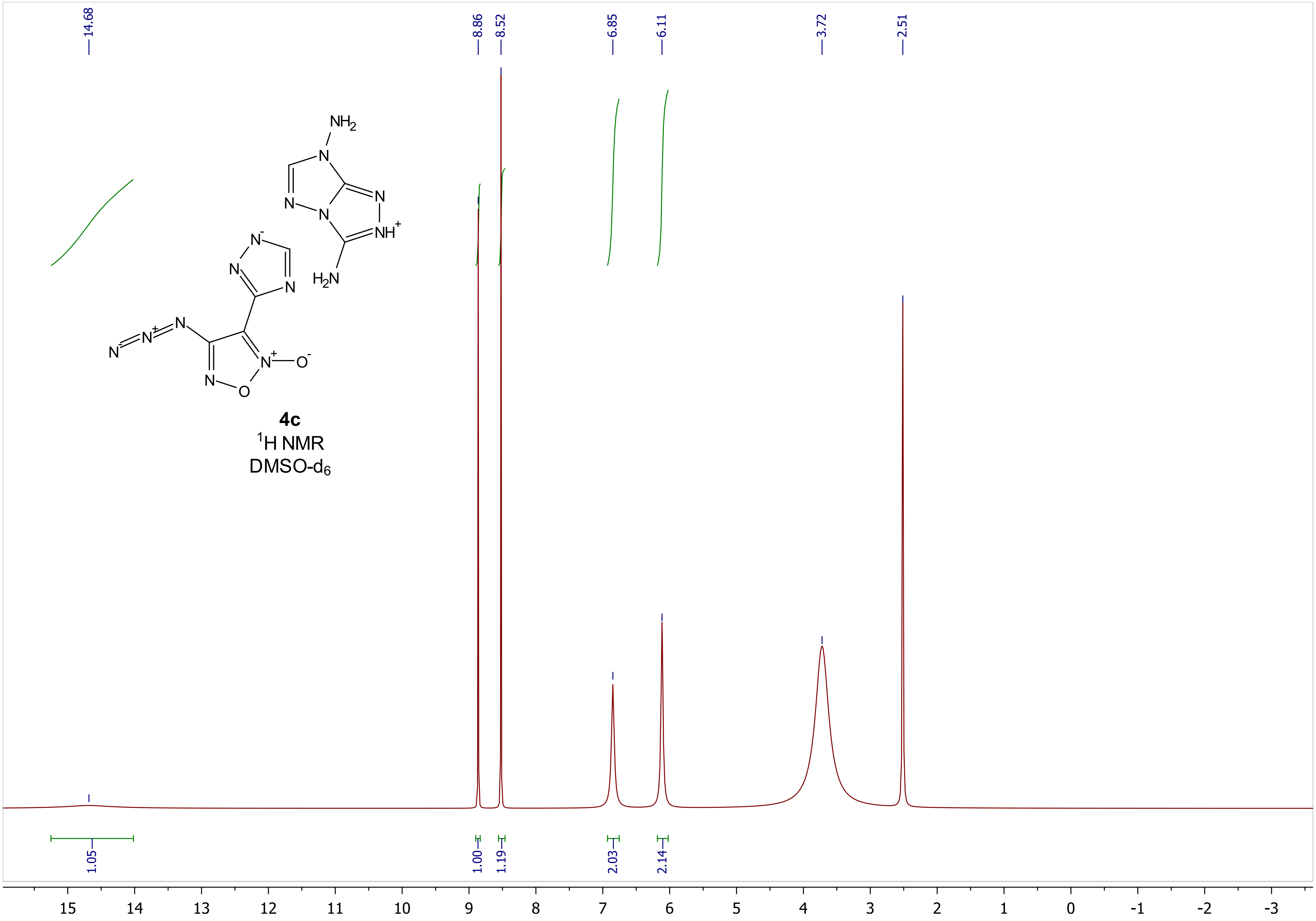

14.68
8.86
8.52
6.85
6.11
3.72
2.51
4c
^{1}H NMR
DMSO-d$_6$
1.05
1.00
1.19
2.03
2.14
15
14
13
12
11
10
9
8
7
6
5
4
3
2
1
0
-1
-2
-3

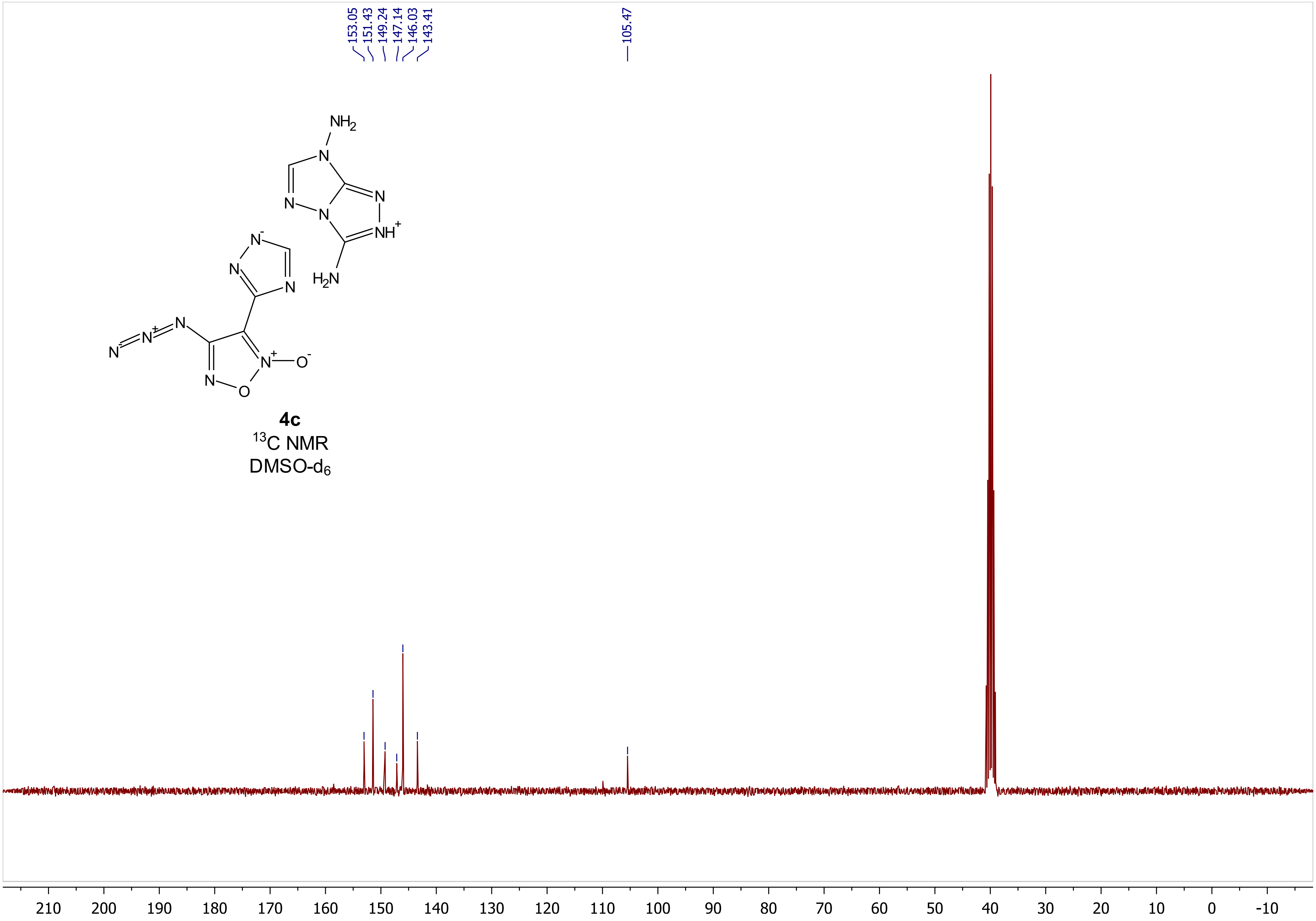
153.05
151.43
149.24
147.14
146.03
143.41
105.47
4c
13C NMR
DMSO-d6
210
200
190
180
170
160
150
140
130
120
110
100
90
80
70
60
50
40
30
20
10
0
-10

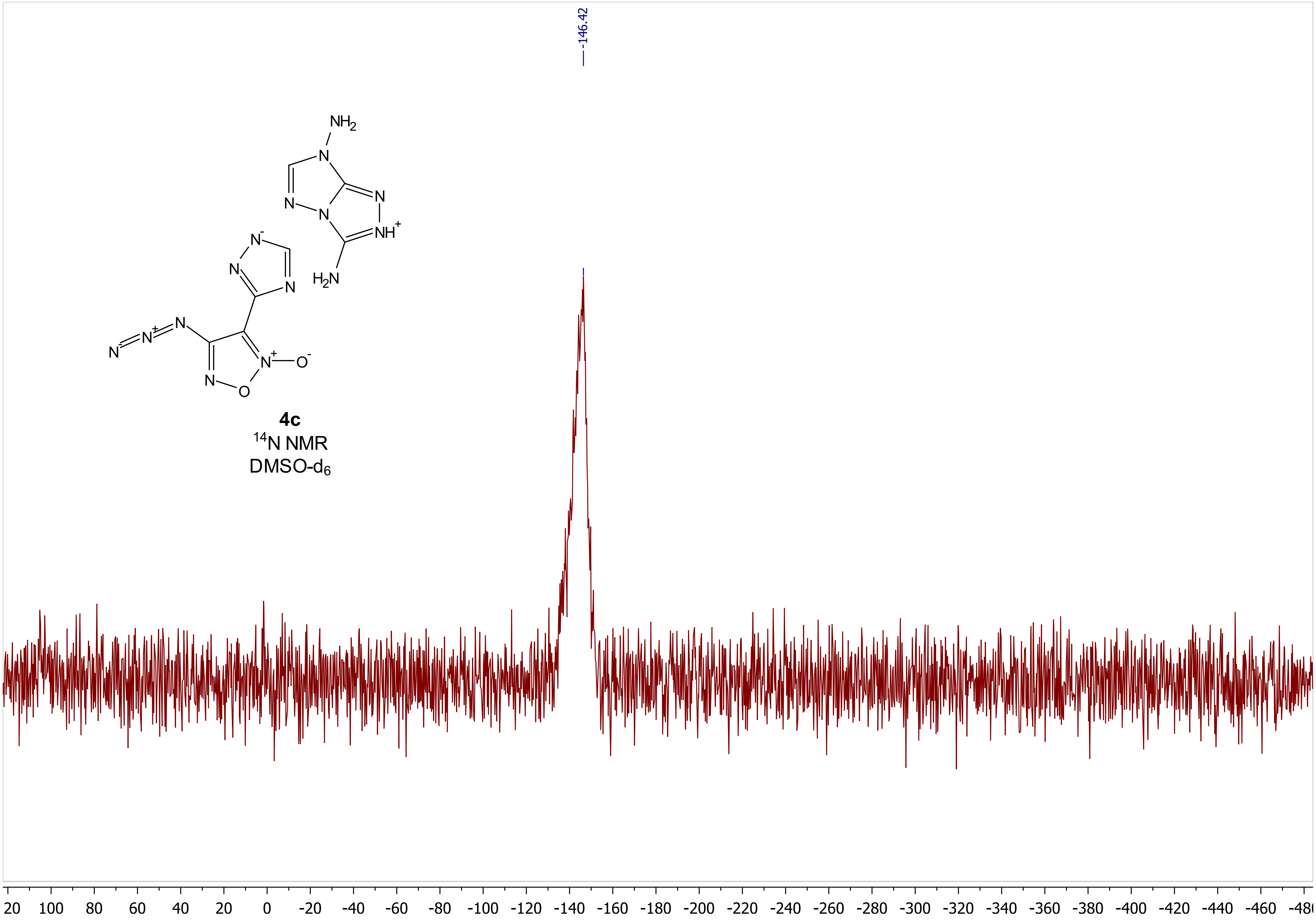
-146.42
NH2
N
N
N
N
NH+
H2N
N-
N
N
N
N+
N
N+
O-
N
O
4c
14N NMR
DMSO-d6
20 100 80 60 40 20 0 -20 -40 -60 -80 -100 -120 -140 -160 -180 -200 -220 -240 -260 -280 -300 -320 -340 -360 -380 -400 -420 -440 -460

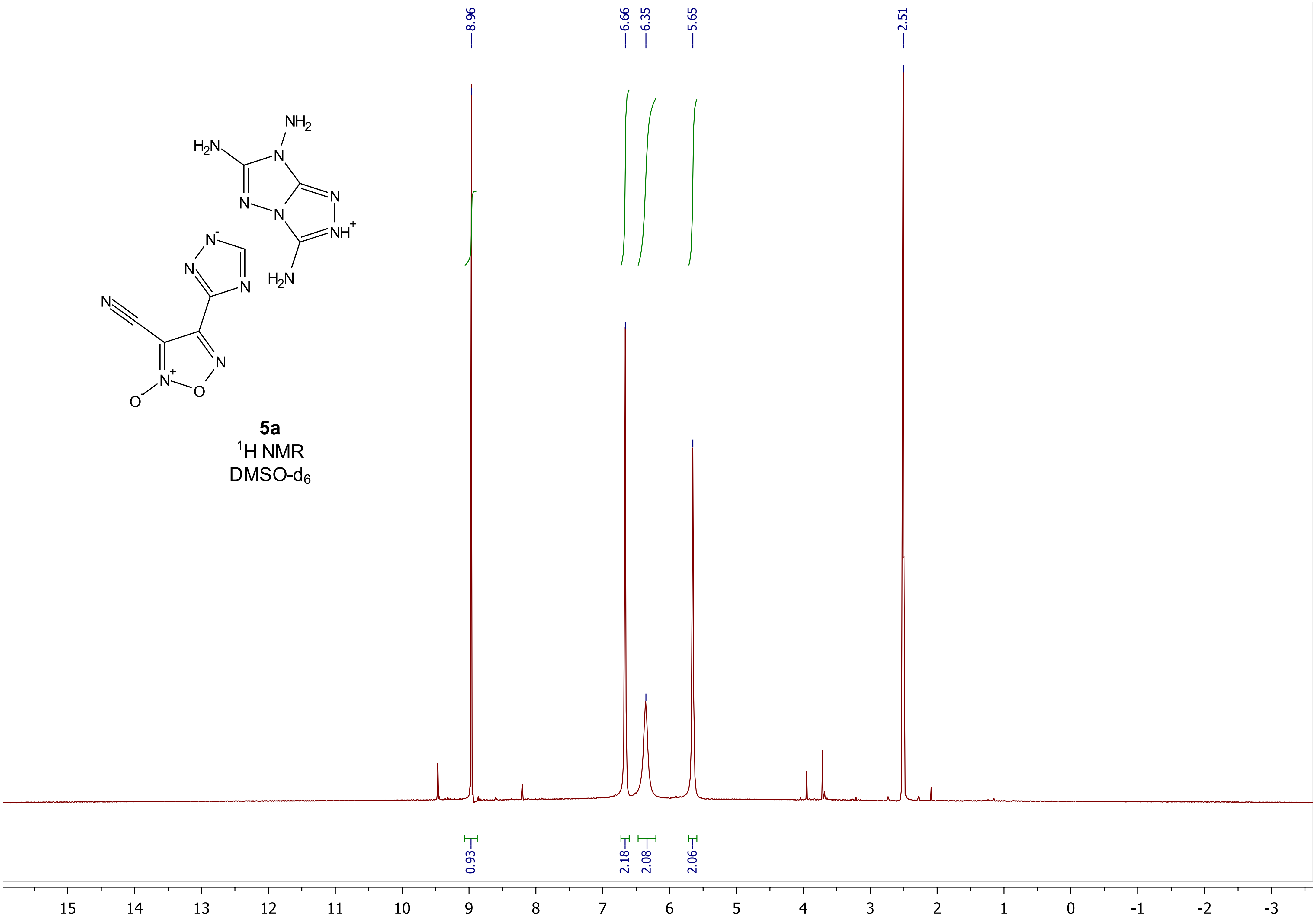

5a
1H NMR
DMSO-d6
8.96
6.66
6.35
5.65
2.51
0.93
2.18
2.08
2.06
15 14 13 12 11 10 9 8 7 6 5 4 3 2 1 0 -1 -2 -3

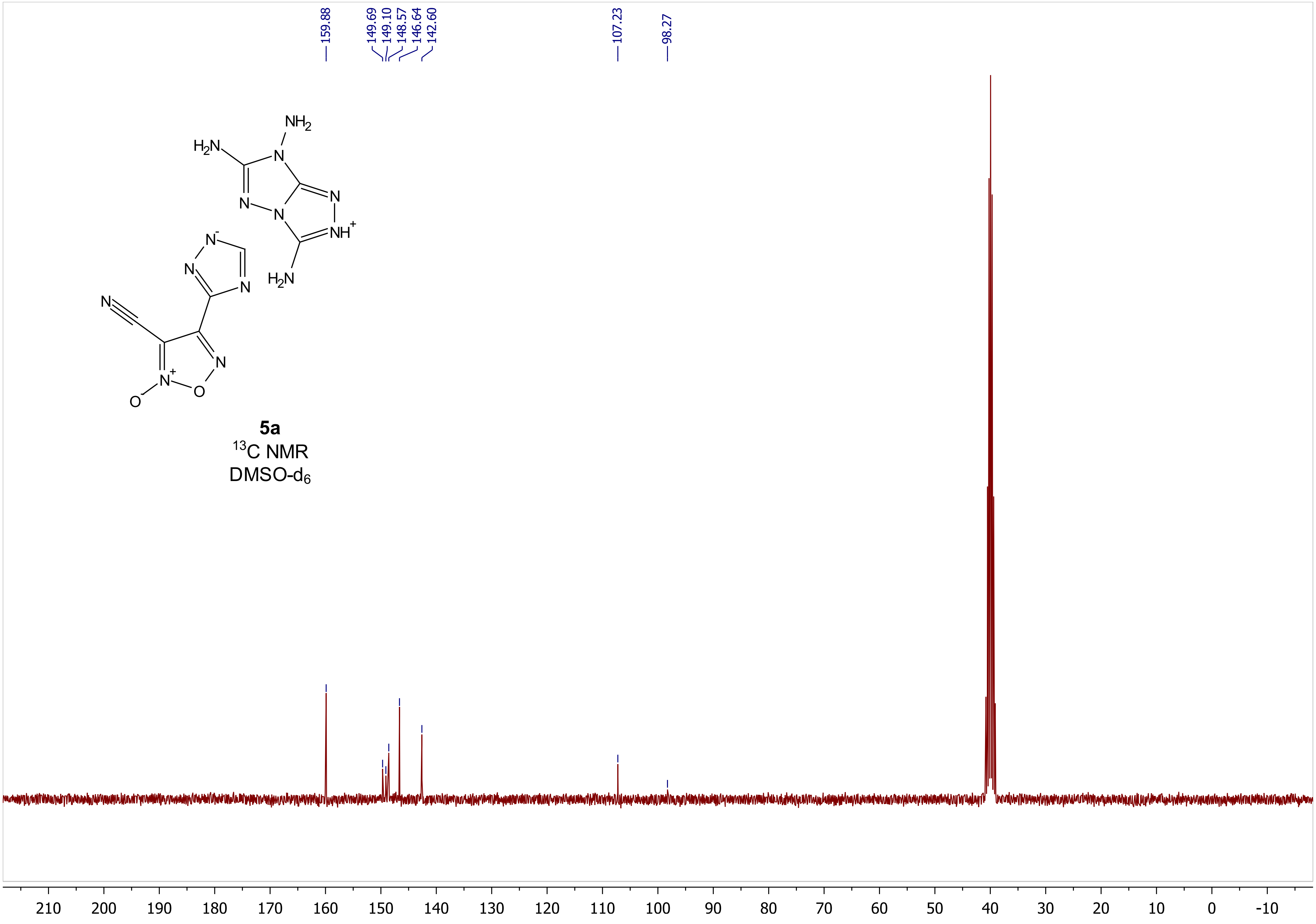
159.88
149.69
149.10
148.57
146.64
142.60
107.23
98.27
5a
^{13}C NMR
DMSO-d_6
210 200 190 180 170 160 150 140 130 120 110 100 90 80 70 60 50 40 30 20 10 0 -10

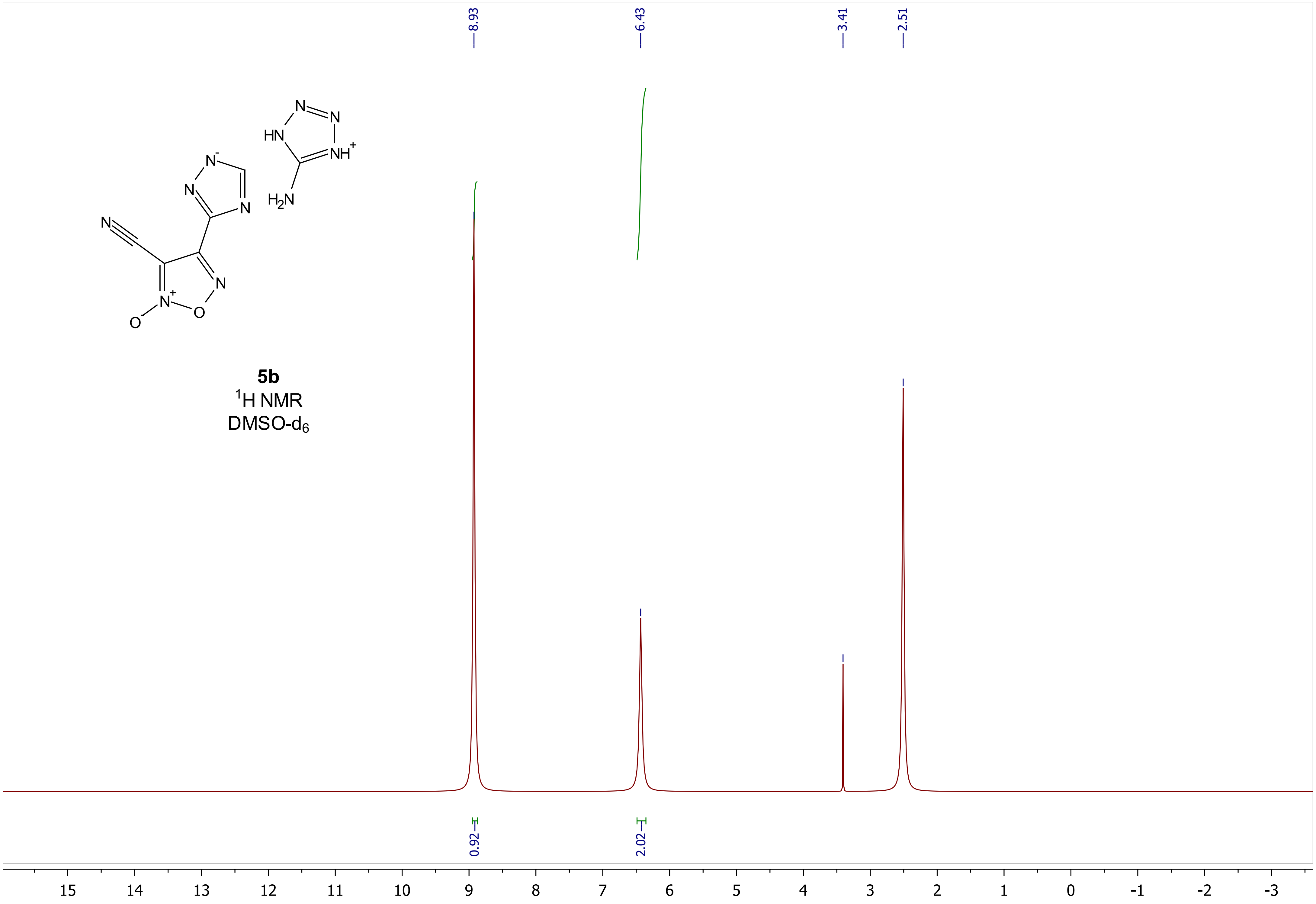

8.93
6.43
3.41
2.51
5b
^{1}H NMR
DMSO-d$_6$
0.92
2.02
15 14 13 12 11 10 9 8 7 6 5 4 3 2 1 0 -1 -2 -3

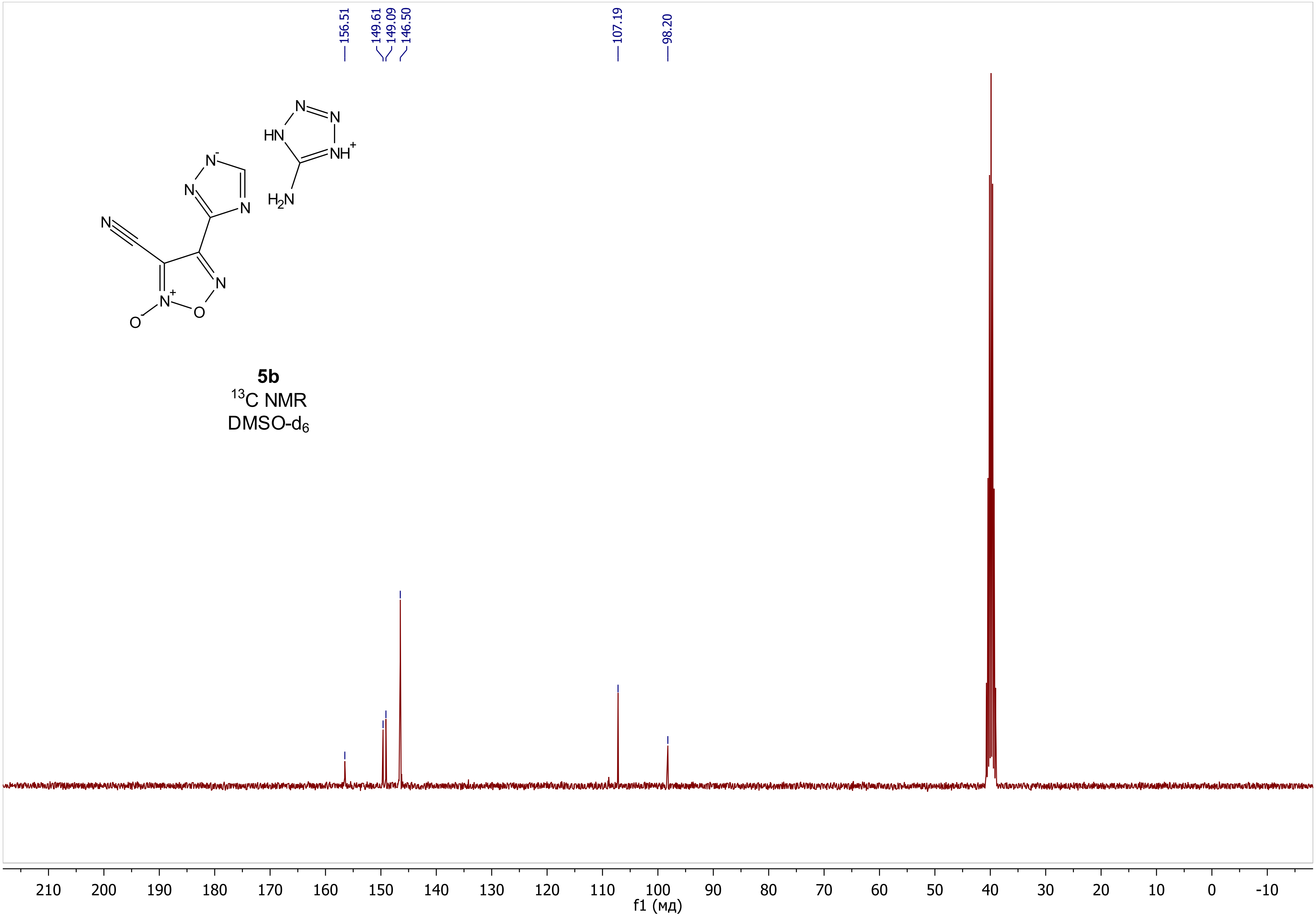
156.51
149.61
149.09
146.50
107.19
98.20
5b
13C NMR
DMSO-d6
210 200 190 180 170 160 150 140 130 120 110 100 90 80 70 60 50 40 30 20 10 0 -10
f1 (мд)

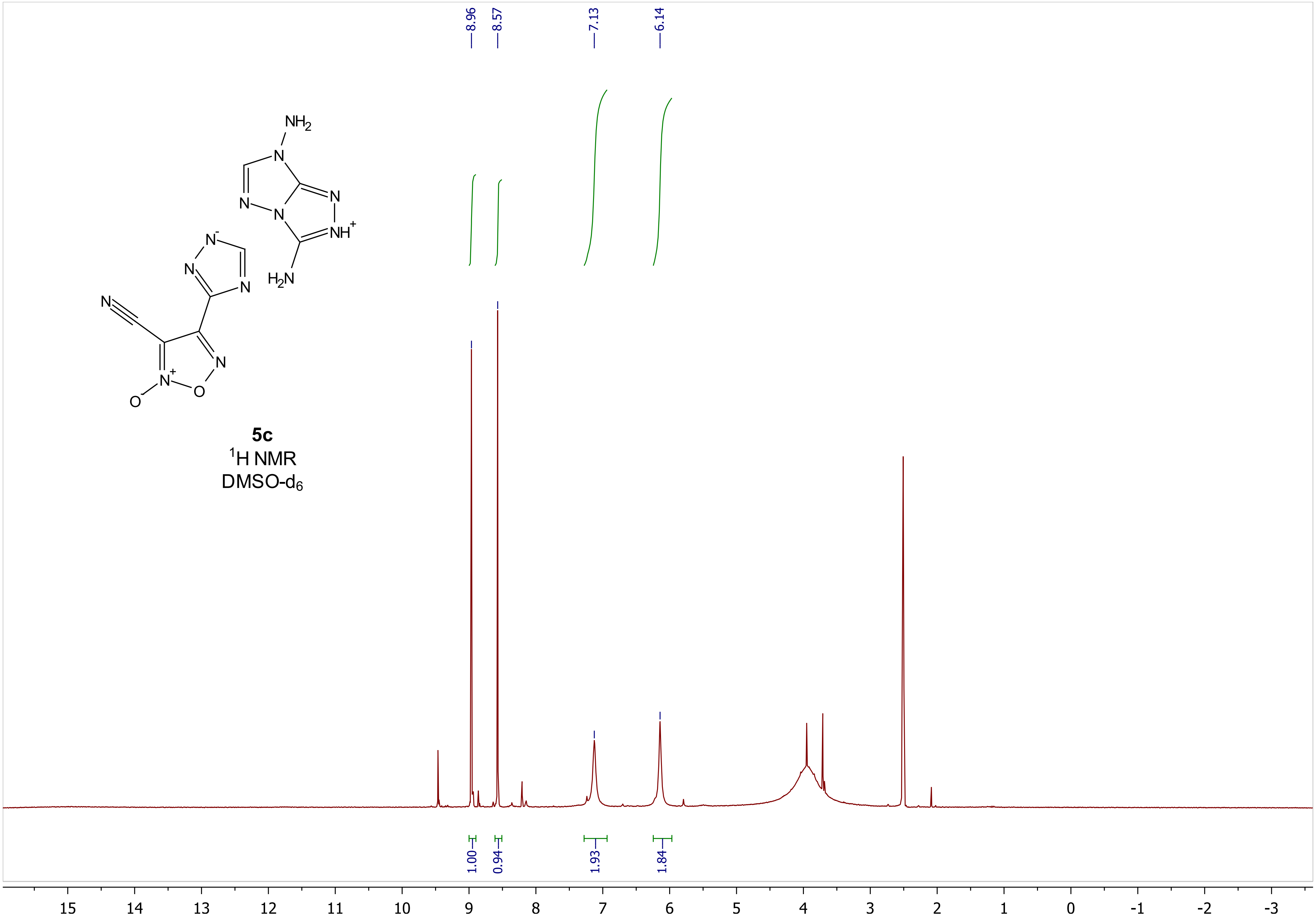

5c
^{1}H NMR
DMSO-d_6
8.96
8.57
7.13
6.14
1.00
0.94
1.93
1.84
15 14 13 12 11 10 9 8 7 6 5 4 3 2 1 0 -1 -2 -3

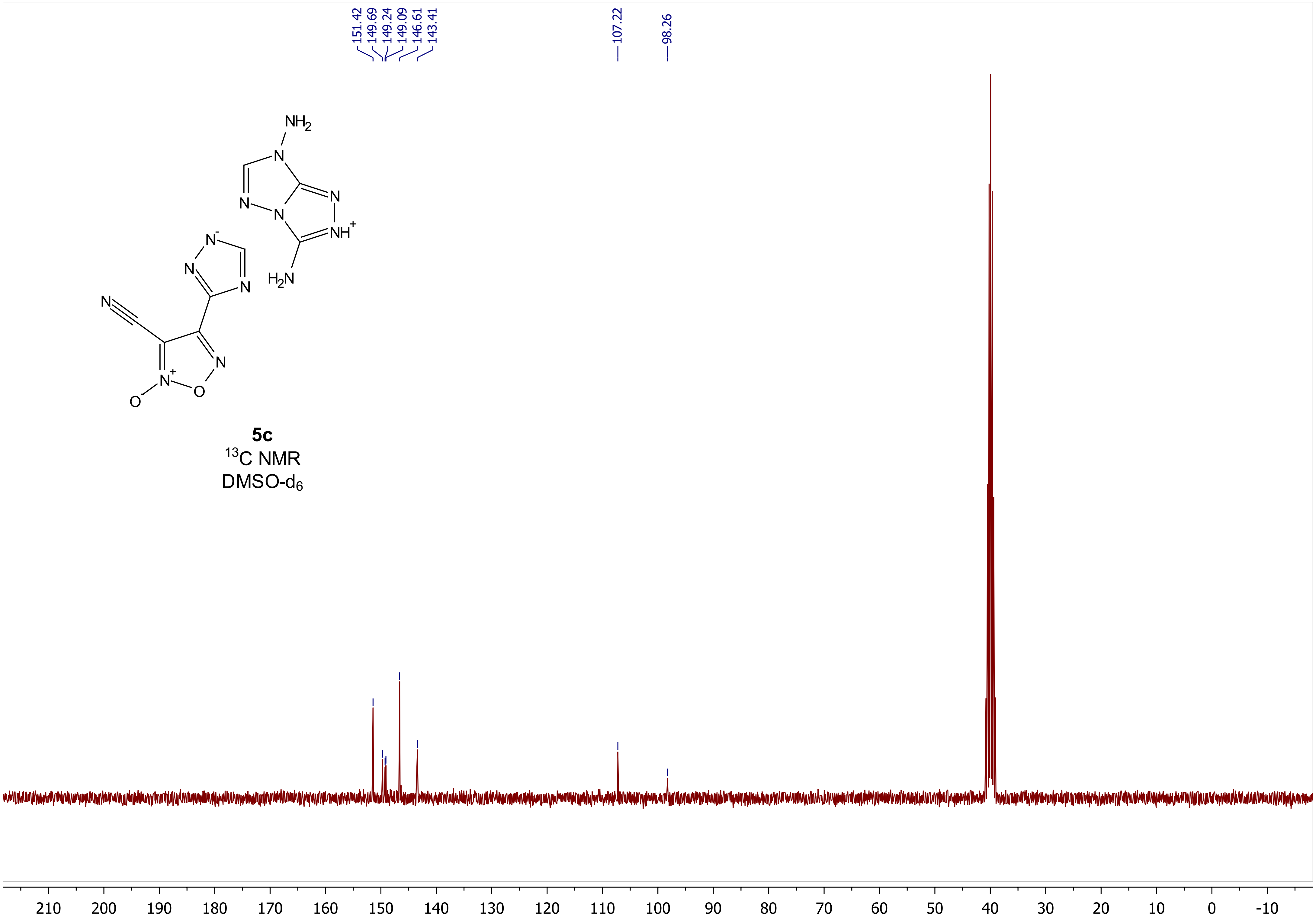

151.42
149.69
149.24
149.09
146.61
143.41
107.22
98.26
NH2
N
NH+
H2N
O
5c
13C NMR
DMSO-d6
210 200 190 180 170 160 150 140 130 120 110 100 90 80 70 60 50 40 30 20 10 0 -10

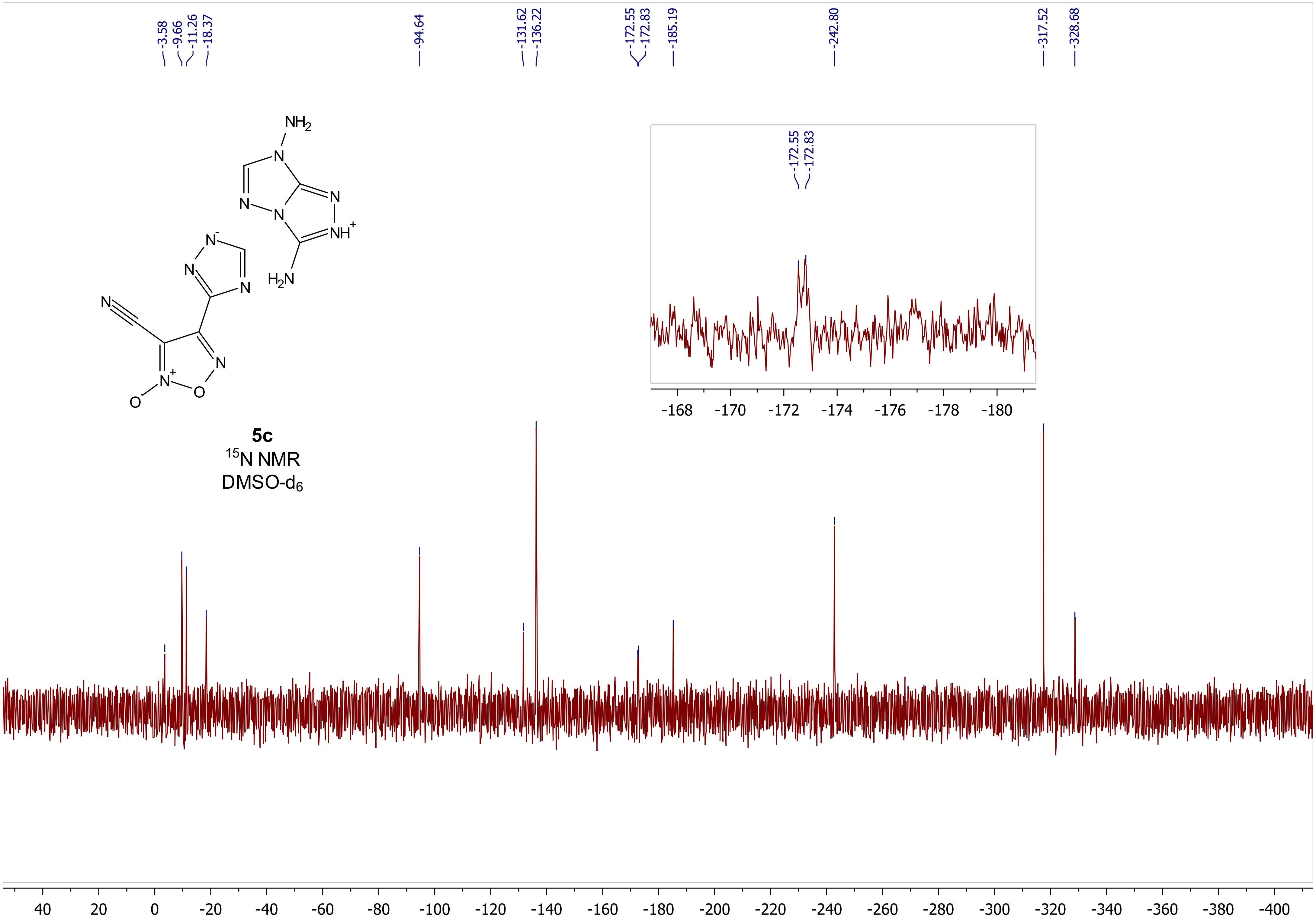

-3.58
-9.66
-11.26
-18.37
-94.64
-131.62
-136.22
-172.55
-172.83
-185.19
-242.80
-317.52
-328.68
5c
15N NMR
DMSO-d6

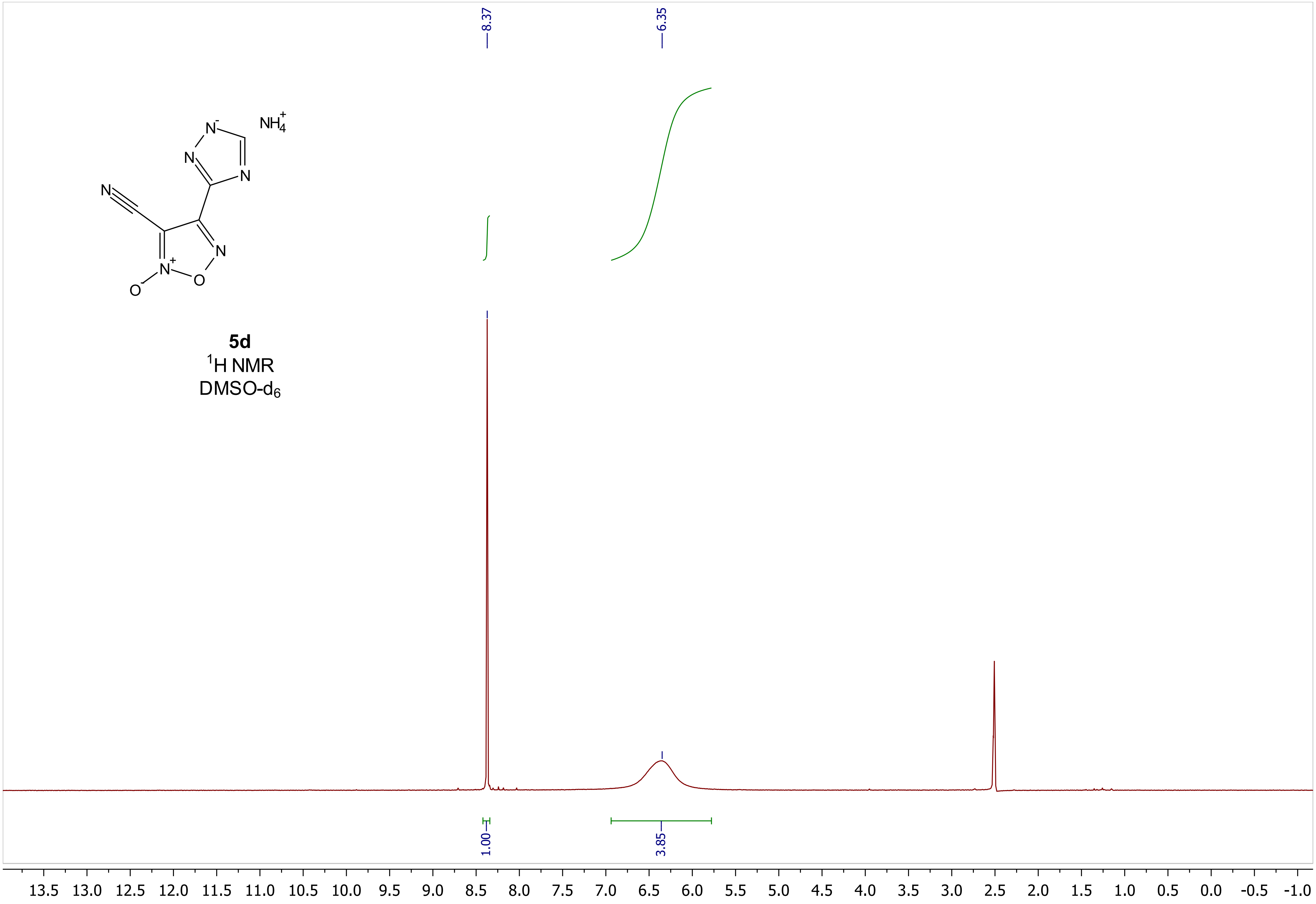
8.37
6.35
NH4+
5d
1H NMR
DMSO-d6
1.00
3.85
13.5 13.0 12.5 12.0 11.5 11.0 10.5 10.0 9.5 9.0 8.5 8.0 7.5 7.0 6.5 6.0 5.5 5.0 4.5 4.0 3.5 3.0 2.5 2.0 1.5 1.0 0.5 0.0 -0.5 -1.0

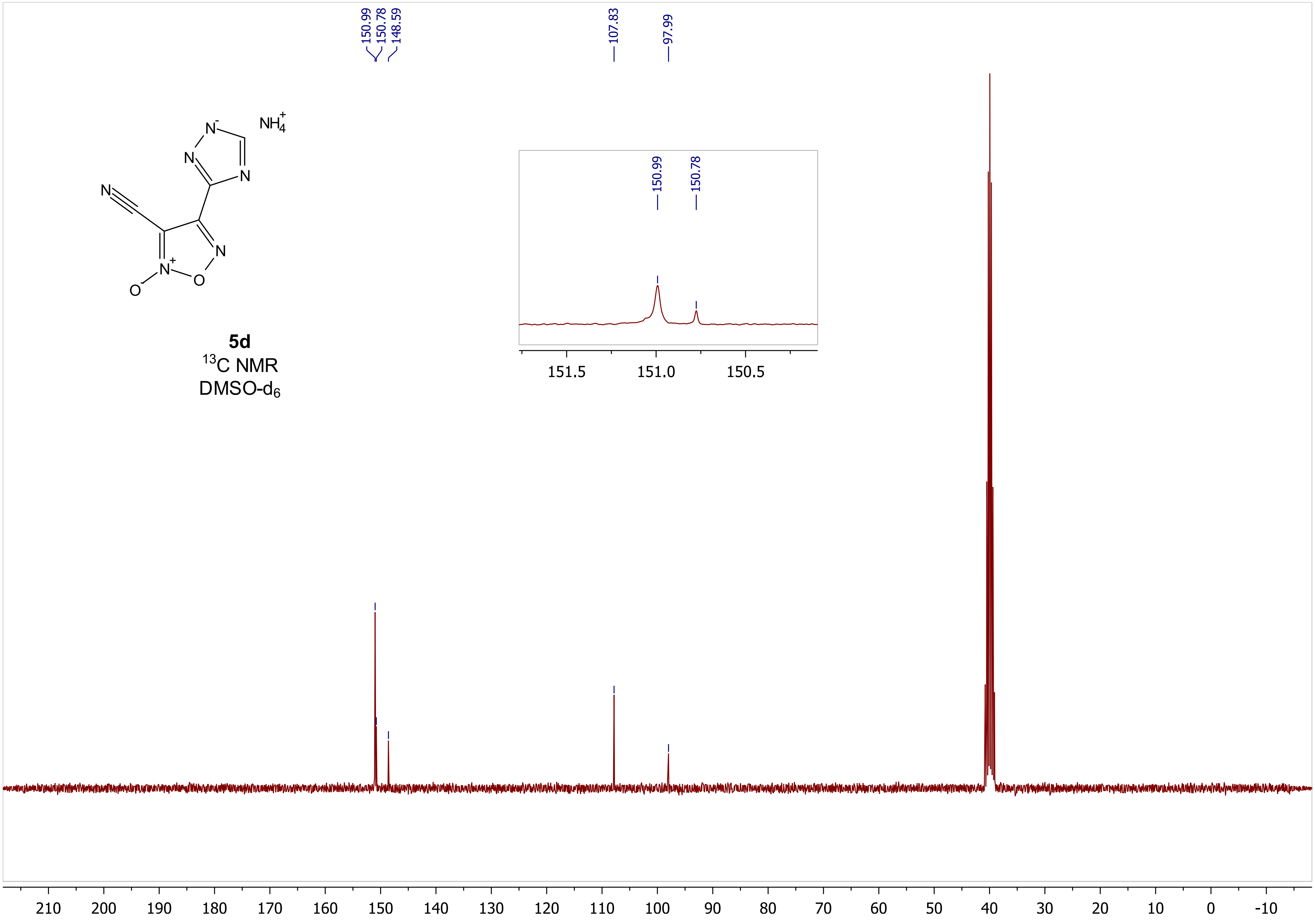

150.99
150.78
148.59
107.83
97.99
NH4+
N
N
N
N
N
N+
O
O-
5d
13C NMR
DMSO-d6
150.99
150.78
151.5
151.0
150.5
210
200
190
180
170
160
150
140
130
120
110
100
90
80
70
60
50
40
30
20
10
0
-10

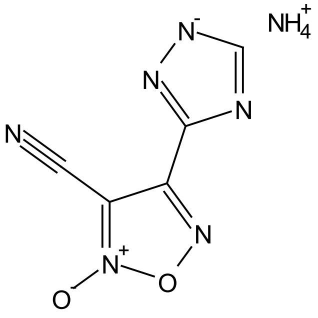

5d
^{14}N NMR
DMSO-d_6

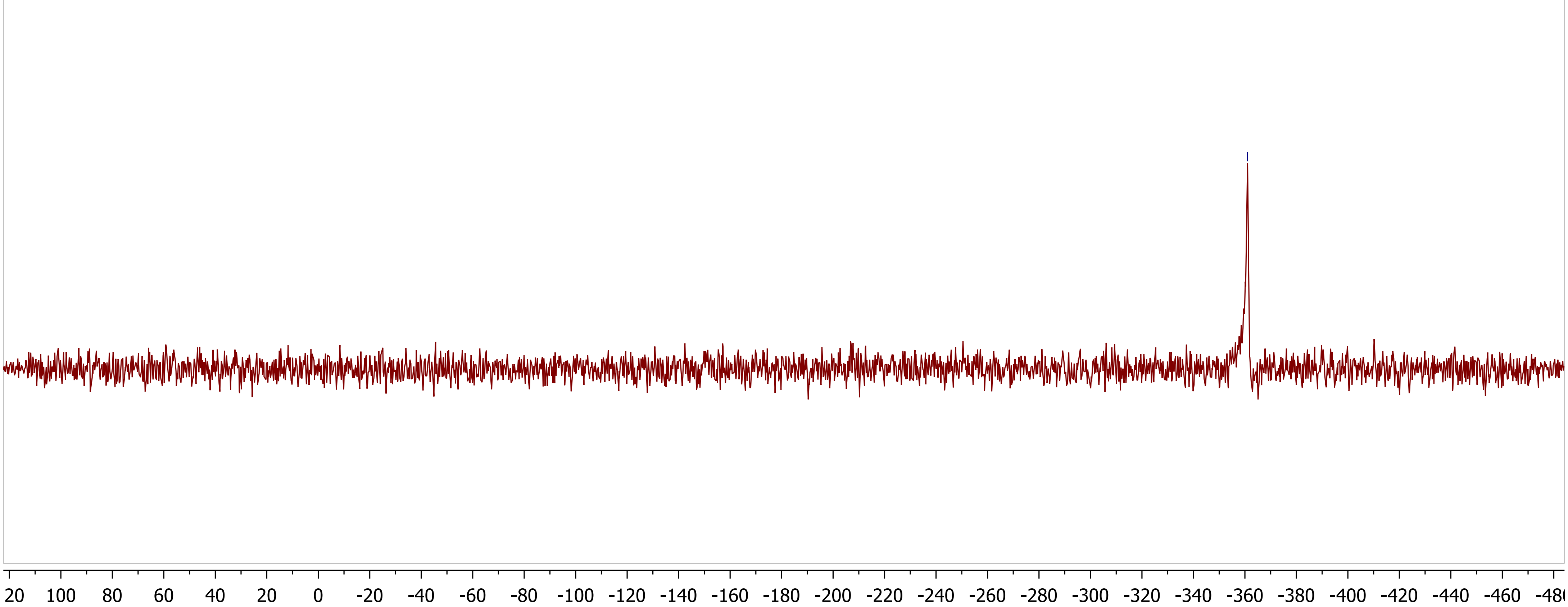

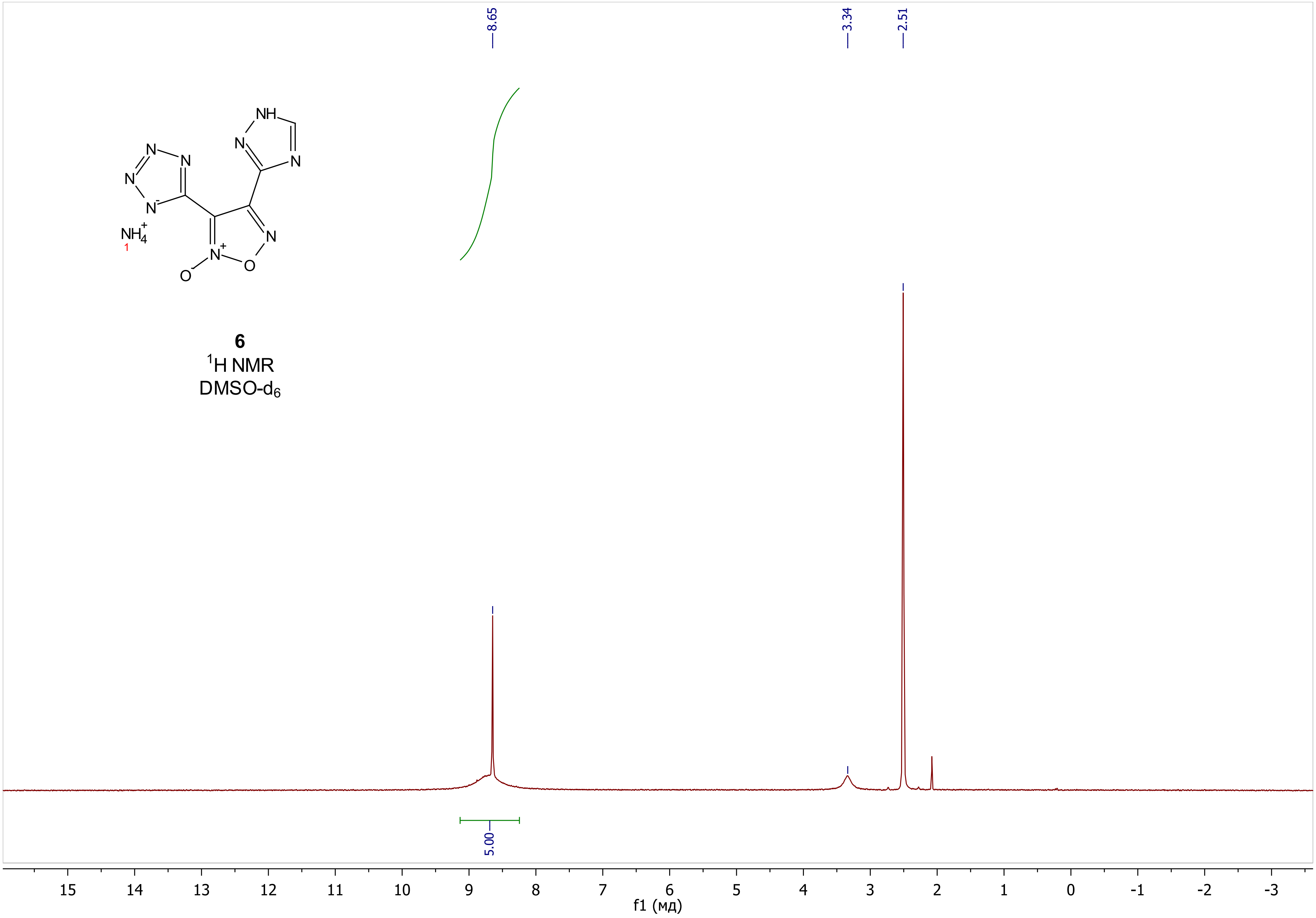

NH
N
N
N
N
N
N-
N
NH4+
1
N+
N
O
O-
6
1H NMR
DMSO-d6
8.65
3.34
2.51
5.00
15
14
13
12
11
10
9
8
7
6
5
4
3
2
1
0
-1
-2
-3
f1 (мд)

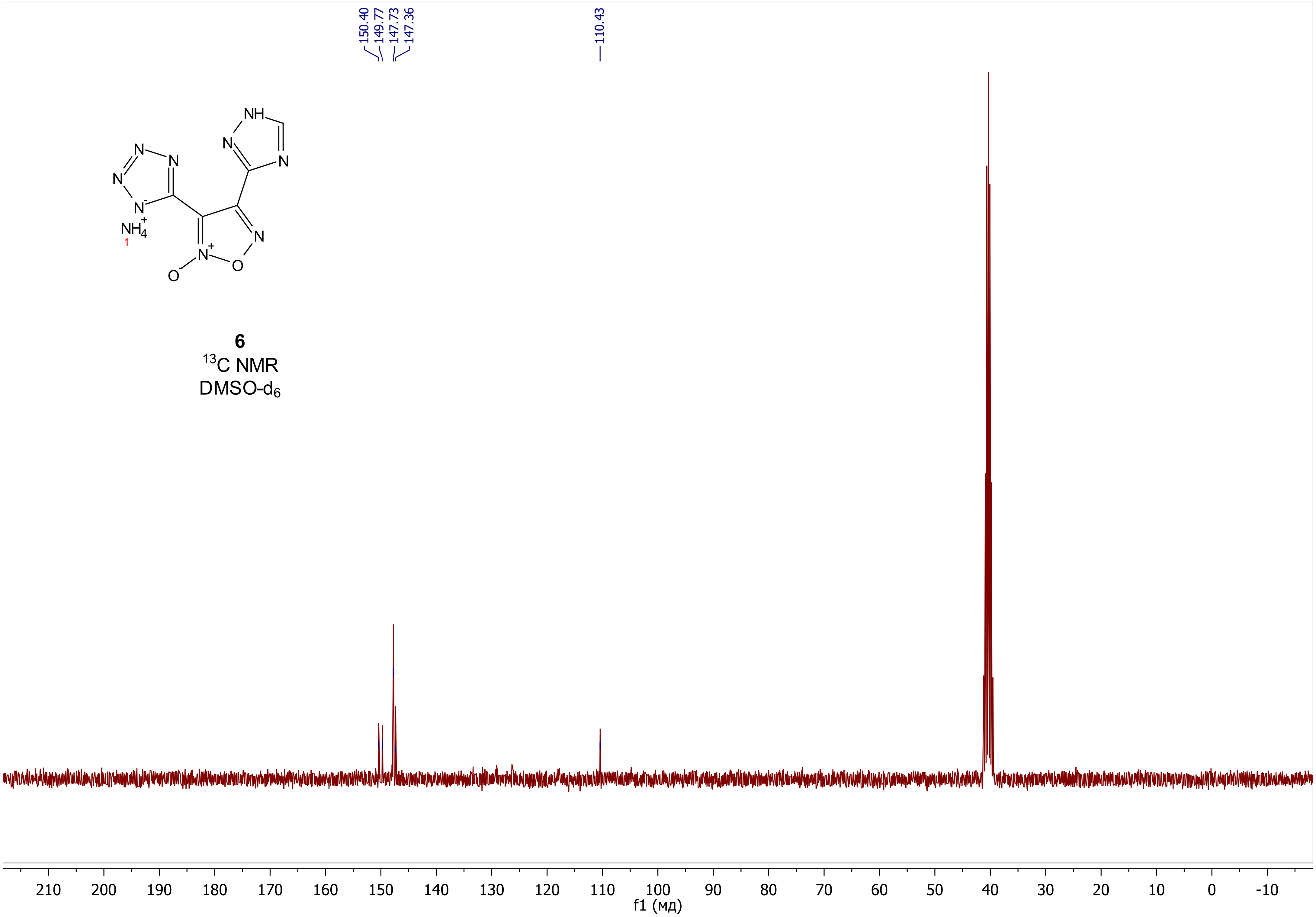
150.40
149.77
147.73
147.36
110.43
NH
N
N
N
N
N
N
NH_4^+
1
N
N
O
O
6
^{13}C NMR
DMSO-d_6
210
200
190
180
170
160
150
140
130
120
110
100
90
80
70
60
50
40
30
20
10
0
-10
f1 (мд)

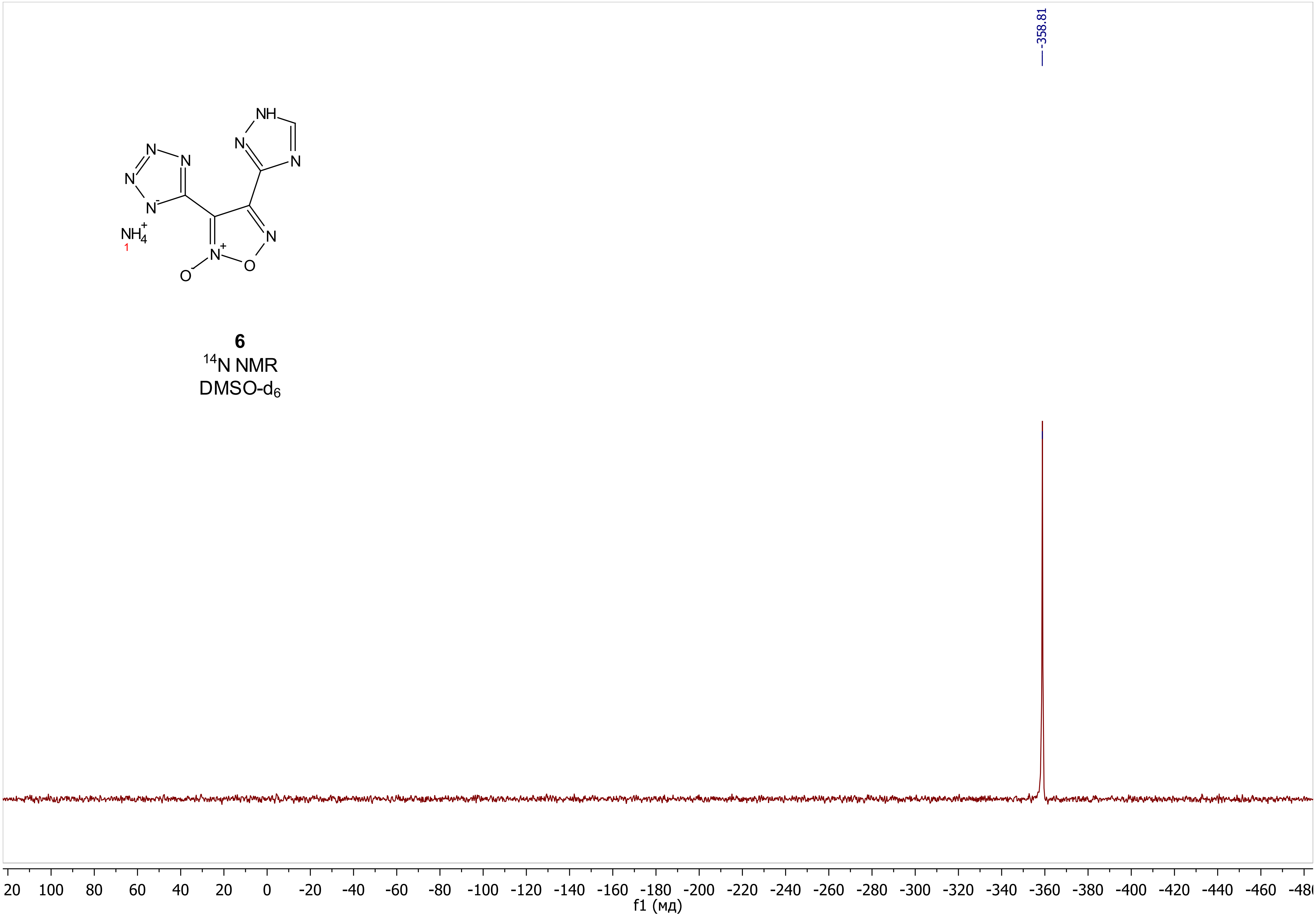
-358.81
N
N
N
N
N
NH
N
N
N
O
O
NH4+
1
6
14N NMR
DMSO-d6
20 100 80 60 40 20 0 -20 -40 -60 -80 -100 -120 -140 -160 -180 -200 -220 -240 -260 -280 -300 -320 -340 -360 -380 -400 -420 -440 -460
f1 (мд)

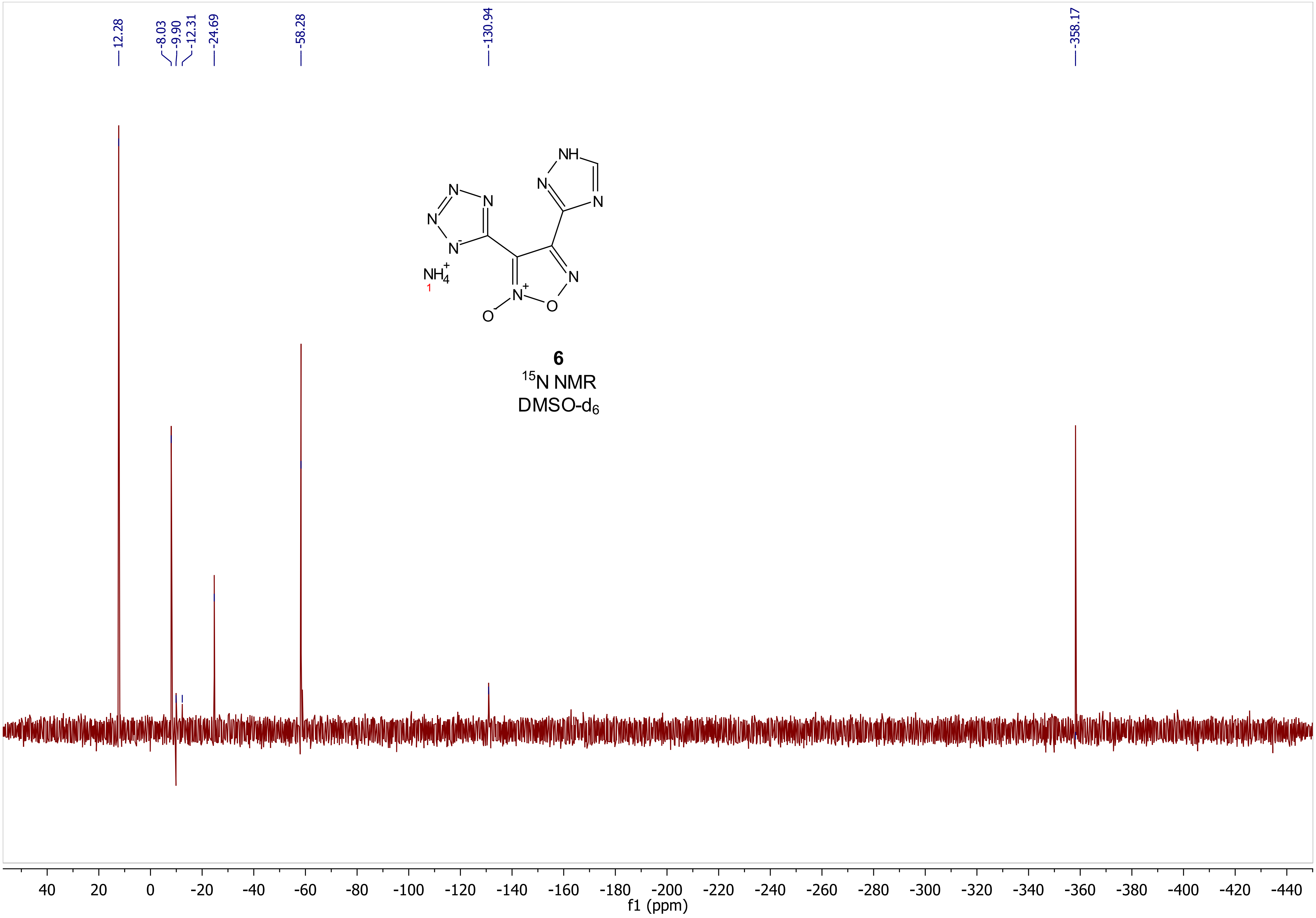

12.28
-8.03
-9.90
-12.31
-24.69
-58.28
-130.94
-358.17
NH
N
N
N
N
N
N
N
N
O
O
NH4+
1
6
15N NMR
DMSO-d6
40
20
0
-20
-40
-60
-80
-100
-120
-140
-160
-180
-200
-220
-240
-260
-280
-300
-320
-340
-360
-380
-400
-420
-440
f1 (ppm)
